# Identification of determinants of differential chromatin accessibility through a massively parallel genome-integrated reporter assay

**DOI:** 10.1101/gr.263228.120

**Published:** 2020-10

**Authors:** Jennifer Hammelman, Konstantin Krismer, Budhaditya Banerjee, David K. Gifford, Richard I. Sherwood

**Affiliations:** 1 Computational and Systems Biology, Massachusetts Institute of Technology, Cambridge, Massachusetts 02139, USA;; 2 Computer Science and Artificial Intelligence Laboratory, Massachusetts Institute of Technology, Cambridge, Massachusetts 02139, USA;; 3 Department of Biological Engineering, Massachusetts Institute of Technology, Cambridge, Massachusetts 02139, USA;; 4 Division of Genetics, Department of Medicine, Brigham and Women's Hospital and Harvard Medical School, Boston, Massachusetts 02115, USA;; 5 Department of Electrical Engineering and Computer Science, Massachusetts Institute of Technology, Cambridge, Massachusetts 02139, USA;; 6 Hubrecht Institute, 3584 CT Utrecht, Netherlands

## Abstract

A key mechanism in cellular regulation is the ability of the transcriptional machinery to physically access DNA. Transcription factors interact with DNA to alter the accessibility of chromatin, which enables changes to gene expression during development or disease or as a response to environmental stimuli. However, the regulation of DNA accessibility via the recruitment of transcription factors is difficult to study in the context of the native genome because every genomic site is distinct in multiple ways. Here we introduce the multiplexed integrated accessibility assay (MIAA), an assay that measures chromatin accessibility of synthetic oligonucleotide sequence libraries integrated into a controlled genomic context with low native accessibility. We apply MIAA to measure the effects of sequence motifs on cell type–specific accessibility between mouse embryonic stem cells and embryonic stem cell–derived definitive endoderm cells, screening 7905 distinct DNA sequences. MIAA recapitulates differential accessibility patterns of 100-nt sequences derived from natively differential genomic regions, identifying E-box motifs common to epithelial–mesenchymal transition driver transcription factors in stem cell–specific accessible regions that become repressed in endoderm. We show that a single binding motif for a key regulatory transcription factor is sufficient to open chromatin, and classify sets of stem cell–specific, endoderm-specific, and shared accessibility-modifying transcription factor motifs. We also show that overexpression of two definitive endoderm transcription factors, *T* and *Foxa2*, results in changes to accessibility in DNA sequences containing their respective DNA-binding motifs and identify preferential motif arrangements that influence accessibility.

Genomic DNA acts as an instruction book for the cellular machinery to carry out functional processes such as RNA production (Sherwood et al. 2014; Miyamoto et al. 2018) and DNA repair (Ball and Yokomori 2011). Some regions of the genome are constitutively used across all cell types for shared housekeeping processes (Cairns 2009; Klemm et al. 2019), whereas other regions are required only in specific cell types (Wang et al. 2012; Liu et al. 2019). One key mechanism used to control which regulatory regions are active is the physical accessibility of chromatin. Because many transcription factors are incapable of binding in inaccessible or “closed” chromatin, the regulation of chromatin accessibility ensures such transcription factors do not bind to extraneous or deleterious locations in the genome.

Transcription factors that interact with closed chromatin are thought to establish the accessibility of cell type–specific regions and initiate cell state change in differentiation (Sherwood et al. 2014; Soufi et al. 2015), cancer (Corces et al. 2016, 2018), and environmental responses (Schick et al. 2015; Lämke and Bäurle 2017) and allow “settler” transcription factors to bind and activate previously inactive genes. Massively parallel reporter assays (MPRAs) (Inoue and Ahituv 2015; White 2015) have been developed to measure the change to gene expression from the action of promoters (Mogno et al. 2013; Grossman et al. 2017) or enhancers (Melnikov et al. 2012; Patwardhan et al. 2012; Kheradpour et al. 2013; Smith et al. 2013; Maricque et al. 2016, 2019) and thus can be used to probe the regulatory code. MPRAs allow for studies into the combinatorial logic of transcription factor action, such as whether specific combinations of transcription factor binding sites must be colocalized for proper gene expression (Smith et al. 2013; Fiore and Cohen 2016; White et al. 2016). However, MPRAs do not measure changes to chromatin accessibility and thus cannot disentangle gene regulation by transcription factors that depend upon changes in local accessibility.

Previous work has indicated specific transcription factor motifs and logic governing chromatin accessibility (Mazzoni et al. 2013; Velasco et al. 2017; Cernilogar et al. 2019), but such effects are difficult to study in a native genomic context, in which motifs are not independent of nonlocal sequence effects. Recent approaches have extended MPRAs to measure nucleosome occupancy via bisulfite treatment (Levo et al. 2017) or MNase-seq (Yan et al. 2018) in yeast. However, bisulfite sequencing requires constrained library design to ensure sufficient CpG sites that act as a substrate for bisulfite conversion, and MNase-seq requires measurement over multiple MNase concentrations to fully measure accessibility (Schwartz et al. 2019). Restriction enzyme strategies have been used to measure nucleosome occupancy and accessibility in yeast (Oberbeckmann et al. 2019) and mouse hepatocyte (Chereji et al. 2019) and stem cells (Soenmezer et al. 2020), and recently, adenine methyltransferase has been used to map nucleosome positioning in human cell lines (Abdulhay et al. 2020; Stergachis et al. 2020). Here, we aim to develop an assay that takes advantage of adenine methyltransferase and restriction enzyme digestion for measuring the local DNA accessibility of genomically integrated large-scale reporter libraries, and probe the regulatory sequence determinants driving differential chromatin accessibility between stem cells and definitive endoderm.

## Results

### Multiplexed integrated accessibility assay measures local accessibility of integrated DNA sequences

In previous work, we used a DNase I cleavage assay, SLOT, to measure chromatin accessibility of a set of DNA sequences integrated into a defined genomic locus (Hashimoto et al. 2016). Although SLOT was able to determine the relative accessibility of classes of DNA sequences, it had poor resolution to measure accessibility of individual DNA sequences, because of the low cleavage probability of DNase I at enzyme concentrations capable of discriminating levels of chromatin accessibility. We hypothesized that we could measure changes in DNA accessibility with higher sensitivity by observing the chromatin accessibility–dependent methylation of *Escherichia coli* adenine DNA adenine methylase (Dam) to the locus, given the high efficiency and stability of Dam methylation in cells (van Steensel and Henikoff 2000) and the known propensity of Dam to methylate more frequently in accessible chromatin (van Steensel and Henikoff 2000; Szczesnik et al. 2019; Abdulhay et al. 2020; Stergachis et al. 2020). We further hypothesized that fusing Dam to retinoic acid receptor-gamma (RAR) would enhance the differential methylation of this RAR-Dam fusion protein at genomic loci with RAR binding motifs, and we make use of a mutant version of Dam methyltransferase shown to display increased signal-to-noise over wild-type Dam (van Steensel and Henikoff 2000; Szczesnik et al. 2019).

**Figure 1.**
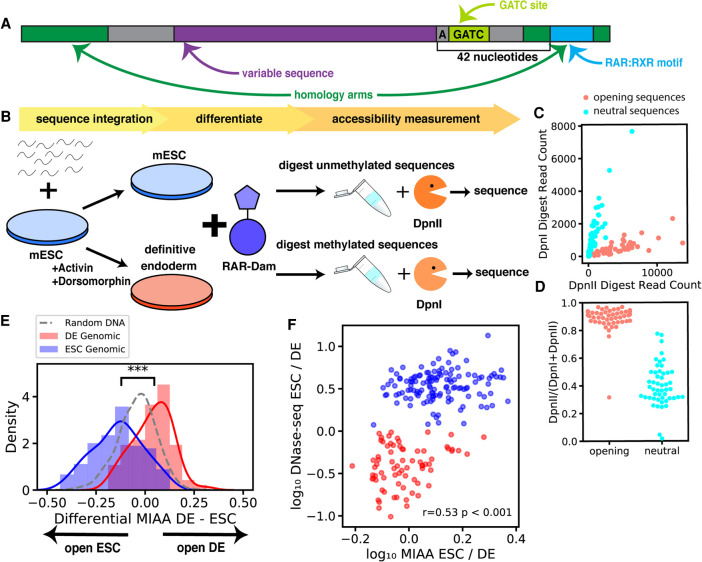
Multiplexed integrated accessibility assay (MIAA) measures local DNA accessibility of synthesized oligonucleotide DNA sequence libraries. (*A*) The MIAA library sequence construct contains a variable DNA sequence, homology arms for CRISPR-mediated HDR integration at a specific genomic locus that includes a binding site for retinoic acid receptor 42 nt downstream from the variable DNA sequence, and GATC site for DNA adenine methylase (Dam) methylation 1 nt downstream from the variable DNA sequence. (*B*) DNA sequences of 150 nt are integrated into ESCs at a designated genomic locus. ESCs are split, and half are differentiated into DE cells. Retinoic acid receptor fused to hyperactivated Dam enzyme results in methylation of DNA sequences that open DNA. DNA is extracted, and half is exposed to DpnII, which cleaves unmethylated sequences, whereas half is exposed to DpnI, which cleaves methylated sequences. Sequences are PCR amplified and sequenced. (*C*) DpnI and DpnII read counts measured from a single DE replicate show difference between designed chromatin opening and neutral DNA sequences. (*D*) Proportion of DpnII read counts measured from a single ESC replicate gives estimate of MIAA openness. (*E*) Genomic sequences are differentially DE accessible or ESC accessible as reported by difference between MIAA Dpn proportion in definitive endoderm compared with ESCs with randomly shuffled DNA control sequences (significance computed by Wilcoxon rank-sum test). (*F*) Differential accessibility as measured by log change in normalized DNase-seq reads and MIAA methylation proportion shows correlation between native differential accessibility and MIAA accessibility. The correlation reported is the Pearson's correlation coefficient (*r*).

We designed a library consisting of 150-nt synthesized oligonucleotides that consist of a 100-nt variable DNA sequence surrounded by a fixed sequence that allows for PCR amplification and contains an Illumina sequencing adapter and a Dam recognition sequence (GATC) (Fig. 1A). For integration, we chose a genomic locus with minimal prior DNase I accessibility proximal to a RAR binding site. To allow inducible expression of RAR-Dam, we integrated a single copy of RAR-Dam with a doxycycline-sensitive promoter into a fixed genomic locus using Cre/LoxP recombination into a mouse embryonic stem cell (mESC) line with constitutive rtTA expression (Mazzoni et al. 2011).

After DNA sequence integration into the mESC cell line, we induce the expression of RAR-Dam and, after 24 h, collect genomic DNA (Fig. 1B). DNA sequences that increase chromatin accessibility should increase adenine methylation of the DNA sequence's GATC site, owing to the combined effect of the preference of Dam methylase to methylate in accessible chromatin, and increased local RAR binding, owing to increased chromatin accessibility. Purified genomic DNA is split it into two pools; one pool is exposed to the restriction enzyme DpnI and the other pool to DpnII, which preferentially cleave methylated and unmethylated GATC sites, respectively. From each pool, we then amplify DNA sequences using a three-step PCR amplification process (Supplemental Fig. S1). First, DNA sequences are amplified by primers outside of the homology arms to ensure only correctly integrated DNA sequences are amplified. Only undigested DNA sequences will be amplified at this step owing to the site of the GATC site of restriction enzyme cleavage between the PCR primers. Then, two additional PCR steps are used to further amplify DNA sequences and add Illumina sequencing adapters for high-throughput sequencing. If a DNA sequence is more accessible, it will have fewer read counts in the DpnI digested pool and more read counts in the DpnII digested pool (Fig. 1C). The proportion of DpnII to DpnI sequencing counts, therefore, represents the impact of that DNA sequence on local DNA accessibility (Fig. 1D). We designate this high-throughput genomically integrated assay of chromatin accessibility the multiplexed integrated accessibility assay (MIAA).

Because our particular interest is in changes to accessibility during differentiation, we differentiated mESCs into definitive endoderm (DE) cells using a well-established differentiation protocol shown to yield >90% DE (Sherwood et al. 2011) before RAR-Dam induction.

We tested a library of 5978 DNA sequences in eight biological replicates (four replicates at sequence integration, each split into two replicates before RAR-Dam activation) for stem cells (ESCs) and four biological replicates (two replicates at sequence integration, each split into two replicates before RAR-Dam activation) for DE cells. To gauge the reliability of MIAA, we included sets of positive and negative control DNA sequences used in our previous work that maximally pack 100-nt variable sequences with DNA sequence motifs shown to have an opening or neutral effect on chromatin by a *k*-mer model trained on DNase-seq (Hashimoto et al. 2016). From MIAA measurements, we found that the Hashimoto et al. positive control DNA sequences yielded significantly higher Dam methylation than the negative control DNA sequences (Fig. 1C,D), with 81%–99% of positive control DNA sequences yielding higher methylation than the average negative control DNA sequence in each replicate (*P* < 0.001 by Wilcoxon rank-sum test for all replicates). We found in comparing control sequences with GC-content in the range of 30%–50%, MIAA replicates had 96%–100% of positive control DNA sequences yielding higher methylation than the average negative control DNA sequence, whereas SLOT had 4.5%–13.6% of positive control DNA sequences yielding higher methylation than the average negative control sequence (Supplemental Fig. S2), suggesting that MIAA provides a marked improvement over SLOT in the measurement of accessibility differences of single DNA sequences in the context of large libraries. Biological replicates of MIAA were also well correlated (Pearson's *r* = 0.5–0.79) (Supplemental Fig. S3).

We note that negative control (accessibility neutral) DNA sequences are still methylated at a rate of 20%–50%. In line with this result, we found ∼20% RAR-Dam methylation in two known native genomic inaccessible chromatin loci as measured by qPCR, compared with 85%–95% methylation at known RAR binding sites (Supplemental Fig. S2). We do not know if this means that RAR-Dam can methylate ∼20% of inaccessible chromatin while it is tightly wound or if the methylation is happening during cell cycle phases when chromatin is accessible. We also found that retinoic acid binding sites within our sequence appeared to have no impact on MIAA results (Supplemental Fig. S4), suggesting that linking RAR to Dam is unlikely to confound our aim of measuring chromatin accessibility.

We separately designed a pilot experiment of 2000 DNA sequences to determine whether MIAA could measure differential chromatin accessibility. First, we ran KMAC, a method for de novo motif enrichment (Guo et al. 2018), on differentially accessible DNase-seq regions using the top 10,000 peaks that were differentially accessible (defined by peak overlap) in DE-accessible or ESC-accessible genomic regions measuring motif enrichment relative to a background the top 10,000 of genomic regions that are DNase accessible in both ESCs and DE. We used a similar methodology to Hashimoto et al. (2016) to maximally pack oligonucleotides with DNA sequence motifs, by starting from a single motif and extending the designed sequence with the highest scoring KMAC motif that overlapped the previous motif by four bases. Our data show that that MIAA was able to separate DNA sequences that were designed to open chromatin in DE cells from those that were designed to open chromatin in ESCs (Supplemental Fig. S5).

We then asked whether MIAA could measure differential accessibility of native genomic sequences. To help identify 100-nt native genomic sequences that were differentially accessible between DE cells and ESCs, we developed a deep learning model trained to predict DNase-accessible regions from underlying DNA sequence and cell type–specific DNase-seq training data. This method, which we call DeepAccess, trains an ensemble of 10 convolutional neural networks on DNase-seq data from ESCs and DE cells to predict whether a 100-nt genomic region is accessible or inaccessible in both cell types that had good performance on held-out genomic regions (for details, see Methods; Supplemental Fig. S6). We tested 213 native DNA sequences that DeepAccess predicted would be differentially accessible between ESCs and DE cells with MIAA, and found that as a group these DNA sequences showed differential accessibility between ESCs and DE cells (Fig. 1E) with a per-sequence effect size that correlates with differential accessibility measured by DNase-seq (Pearson's *r* = 0.53; *P* < 0.001) (Fig. 1F). Although statistically significant as a group, only 78% of the native genomic DNA sequences recapitulated the differential accessibility of the native loci from which they were derived by having both higher DNase-seq read counts and greater MIAA-measured accessibility in one cell type over the other. These 100-nt endogenous sequences were selected by DeepAccess from DNase-seq accessible regions that can be kilobases in length, so we hypothesize that sequences for which we did not observe differential accessibility may not contain all of the binding elements controlling accessibility of the native locus or may rely on either local or distal interactions with chromatin that were not recapitulated at our genomic integration site. The observed correlation in differential accessibility between DNase-seq and MIAA suggests that a 100-bp sequence transplanted into a specified locus can retain a substantial amount of the information required to encode a particular level of chromatin accessibility (Fig. 1F).

We also included in our library a randomly shuffled nucleotide counterpart for each DNA sequence in order to account for any potential effects of nucleotide composition. We found that most native genomic sequences that were more accessible in ESCs than in DE cells had similar accessibility in ESCs compared with randomly shuffled DNA controls but had lower accessibility in endoderm compared with shuffled control DNA sequences (Supplemental Fig. S7). We hypothesized that these DNA sequences contain motifs that result in decreases in accessibility in DE cells. We performed motif enrichment (for details, see Supplemental Methods) on these DNA sequences and found that 98% (compared with 0% of endoderm native sequences) contained a match to the ZEB2 motif (Supplemental Fig. S7), a known transcriptional repressor that has been implicated in early gastrulation by repression of CDH1 (also known as E-cadherin) (Acloque et al. 2017), suggesting that the DeepAccess-selected ESC sequences were selected based on an endoderm-specific repressor of chromatin accessibility. In contrast, none of our DeepAccess-selected native genomic sequences contained motifs for the known ESC reprogramming factors POU5F1, SOX2, or KLF4 (Soufi et al. 2015), which we would expect to increase chromatin accessibility in ESCs.

To investigate why DeepAccess chose ESC native genomic sequences that contain ZEB2 motifs over known reprogramming factors, we compared DeepAccess-predicted differential accessibility for ChIP-seq sites for the known pluripotency factors POU5F1, SOX2, and KLF4, which contained their DNA-binding motifs along with ZEB2 genomic motif instances, and found that although the known pioneer transcription factor motifs had positive effects on ESC accessibility, ZEB2 motifs had the strongest predicted effect on differential accessibility by the presence of the motif causing a decrease in predicted accessibility in DE cells (Supplemental Fig. S7). ZEB2 binding sites were also enriched in ESC-specific genomic accessible regions with 14% containing a ZEB2 motif relative to 9% in endoderm-specific accessible regions (*P* < 0.001 by hypergeometric test). In comparison, 12% of genomic ESC-specific accessible regions contained a SOX2 motif, 6% contained a POU5F1 motif, and 6% contained a KLF4 motif. KEGG biological pathway analysis of ZEB2 motif sites in ESC-accessible regions showed an enrichment of motif sites proximal to genes regulating pluripotency of ESCs (*P* < 0.001), including the key pluripotency regulators KLF4, SOX2, and NANOG, a finding that is consistent with a model of ZEB2 repression of pluripotency during DE differentiation (Stryjewska et al. 2017). The ZEB2 motif is similar to motifs of other E-box epithelial–mesenchymal transition driver transcription factors such as ZEB1, SNAI1, SNAI2, and TWIST1 (Stemmler et al. 2019), all of which are expressed during ESC differentiation to endoderm. We note that subsequent MIAA libraries described in this paper show that DNA sequences containing POU5F1, SOX2, and KLF4 motifs do yield ESC-enriched accessibility. Overall, we find that 100-nt DNA sequences extracted from genomic regions with differential chromatin accessibility recapitulate this differential accessibility when transplanted to a fixed chromatin locus.

### DNase-seq analysis identifies motifs driving cell type–specific accessibility

**Figure 2.**
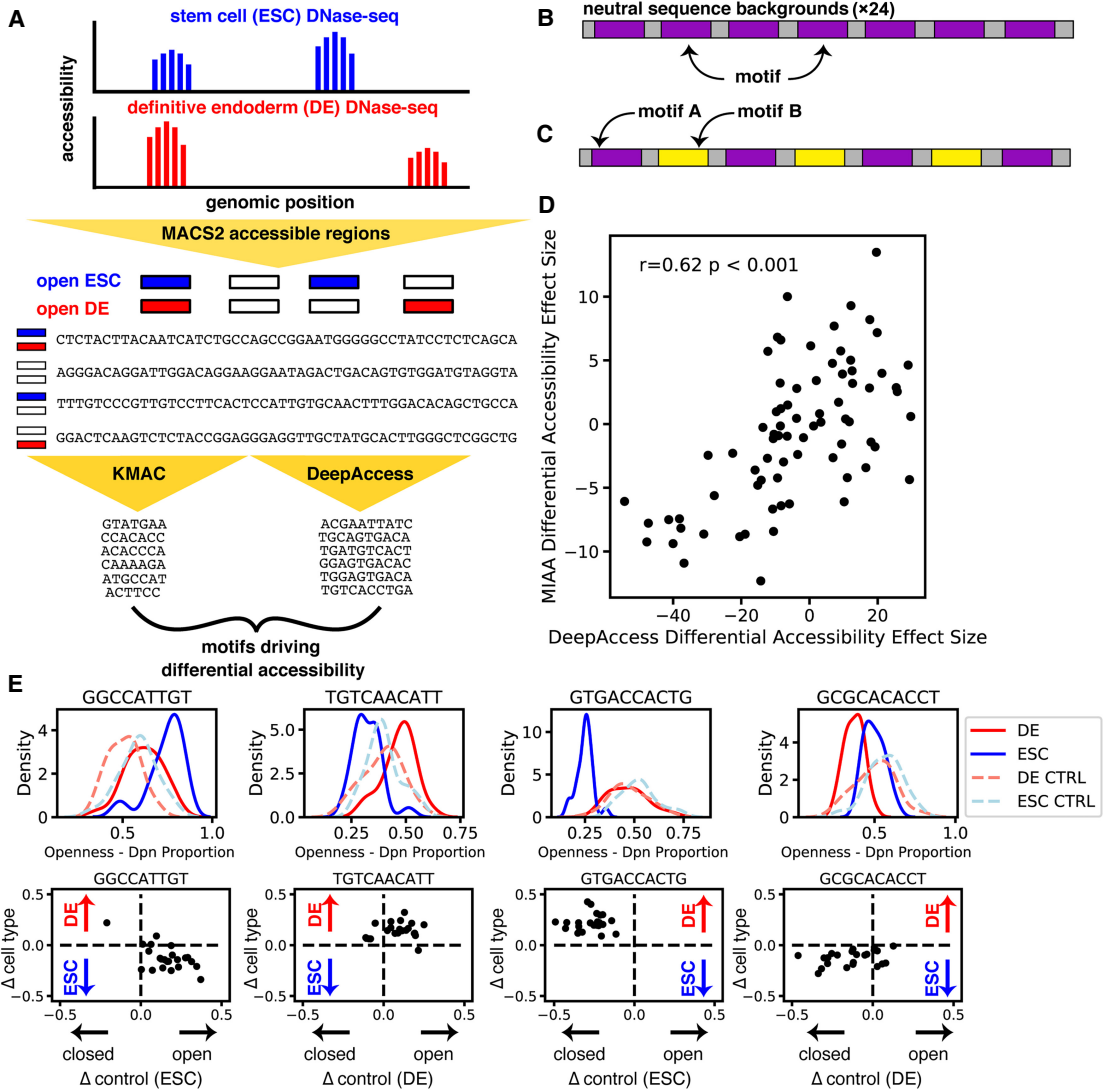
Differentially accessible motif generation from DNase-seq data validated by MIAA. (*A*) DNase-seq accessible regions called with MACS2 and 100-nt sequences extracted centered at narrow peak. KMAC and DeepAccess were applied to extract significant motifs potentially driving differential accessibility between ESCs and endoderm. (*B*) DNA sequences were designed using seven instances of each motif at the same locations in each DNA sequence inserted into 24 100-nt neutral sequence backgrounds, as well as pairs of motifs (*C*). (*D*) Predictions from DeepAccess for differential accessibility replicate experimental results (effect size by paired *t*-test between ESC and DE measurements). The correlation reported is the Pearson correlation coefficient (*r*). (*E*) Motif sequences show differential accessibility via opening ESC, opening endoderm, closing ESC, and closing endoderm (*left* to *right*). (*Top* row) Distribution of MIAA-measured accessibility in ESCs and DE cells for KMAC- or DeepAccess-generated motif, tested over 24 neutral sequence backgrounds and randomly shuffled DNA controls (CTRL). (*Bottom* row) Measurements for a particular DeepAccess or KMAC motif. Each dot represents a single neutral background. The *y*-axis is the difference between endoderm and ESC accessibility, and the *x*-axis is the difference between each DNA sequence and its shuffled control. The cell type in which control measurement is made is in parentheses.

We then hypothesized that we could identify and confirm with MIAA motifs that control chromatin accessibility in a cell type–specific manner through a set of synthetic, designed DNA sequences. By using cell type–specific DNase-seq data, we extracted short (8- to 12-nt) DNA sequence motifs that we hypothesized would cause differential accessibility using two methods (Fig. 2A). First, we used the motifs that were derived from de novo motif discovery by running KMAC on ESC differentially accessible and DE differentially accessible genomic regions. Second, we used DeepAccess to obtain hypotheses about which motifs were most responsible for differential accessibility between DE cells and ESCs (for details, see Supplemental Methods). Unlike KMAC's pure enrichment approach, DeepAccess is able to learn nonlinear relationships between sequence motifs for predicting accessibility. From our set of motif hypotheses from both methods, we designed synthetic DNA sequences with either seven instances of one motif (Fig. 2B), which we call *motif sequences*, or two different motifs (Fig. 2C), which we call *motif pair sequences*, inserted into 24 fixed sequence backgrounds of varied GC-content. Fixed background sequences were previously measured to have a neutral impact on cell type–specific accessibility with MIAA (see Methods for details). We chose to pack each DNA sequence with the maximum number of motifs (54%–84% of the positions in each DNA sequence are part of a motif) while leaving space for sequence variation. For each DNA sequence, we also included a control in which the nucleotides are randomly shuffled to observe the influence of nucleotide content alone.

To determine whether DeepAccess was able to predict the effects of motif sequences or motif pair sequences, we compared the DeepAccess-predicted effect size of each motif or motif pair on differential accessibility to the equivalent MIAA measurement. We found that DeepAccess results are correlated (Pearson's *r* = 0.62; *P* < 0.001) with MIAA-measured differential accessibility (Fig. 2D). However, we found that DeepAccess failed to perform well in predicting paired effects between DNA sequences and shuffled controls (ESC Pearson's *r* = 0.24; DE Pearson's *r* = 0.42) (Supplemental Fig. S8), which we hypothesize is the result of overconfidence of neural networks on out-of-distribution inputs (Nguyen et al. 2015; Moosavi-Dezfooli et al. 2016), because the network had not seen the shuffled control DNA sequences during training. We tested for statistically significant differential accessibility of our motifs and motif pairs by first performing paired tests between MIAA openness in ESCs and DE cells and then performing paired tests between DNA sequences and shuffled controls under a Benjamini–Hochberg multiple hypothesis correction at a false-discovery rate of 0.05 (for details, see Supplemental Methods). Out of 38 tested motif sequences, 20 induced differential accessibility, and out of 38 motif pair sequences, 26 induced differential accessibility. We also found these results to be largely consistent across a secondary closed integration locus (Supplemental Fig. S9). Thus, MIAA was able to confirm that motifs identified using DeepAccess are able to result in observable changes to accessibility both between cell types and compared with shuffled control sequences (Fig. 2E).

Out of the 46 motif or motif pair sequences that induced differential accessibility across cell types and were compared with shuffled control sequences as measured by MIAA, DeepAccess predicted the correct direction of differential accessibility between the two cell types in 76% (35/46) of cases (Supplemental Table S1). In comparing results from DeepAccess to KMAC, we found only 32% (8/25) of our KMAC motifs or motif pairs were differentially accessible compared with 74.5% (38/51) of DeepAccess (Supplemental Table S1), indicating our DeepAccess approach was successful in identifying motifs driving differential accessibility.

### GC-content and transcription factor binding motifs control accessibility

**Figure 3.**
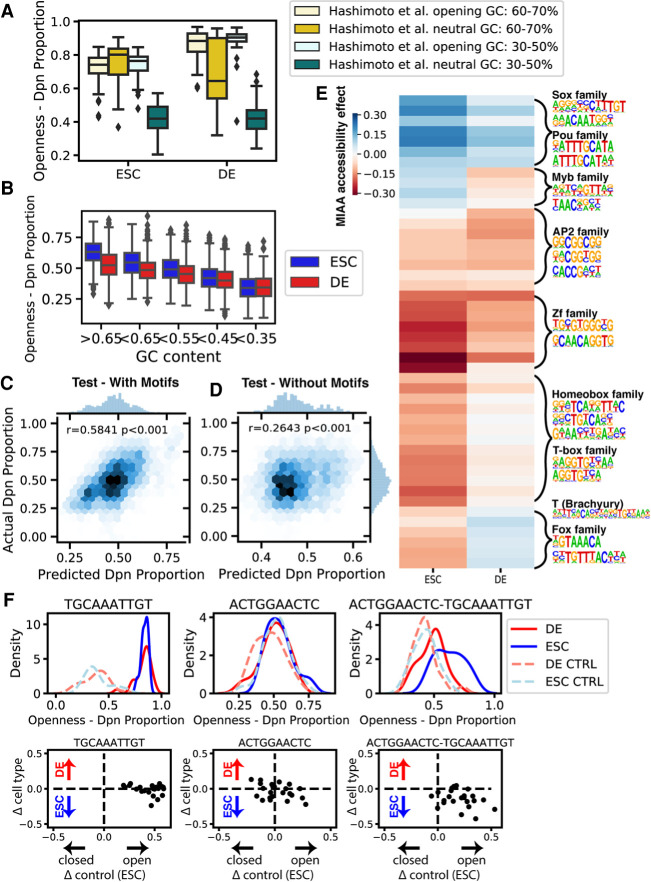
MIAA identifies global influence of GC-content and differentially accessible motifs. (*A*) GC-content observed to be correlated with accessibility in both stem and endoderm cells from positive (Hashimoto et al. opening) and negative (Hashimoto et al. neutral) control sequences. (*B*) GC-content correlated with accessibility in random DNA sequences. A regression model was trained on MIAA Dpn proportions with GC-content, replicate, and cell type–specific effects of 20 motifs and 26 motif pairs as features, and predicts well on (*C*) held-out test data (*n* = 4404) and performs significantly better than (*D*) a model trained without motif variables (adjusted R-squared motif model = 0.398; adjusted R-squared no motif model = 0.095). The correlation reported is the Pearson correlation coefficient (*r*). (*E*) Regression weights of individual motifs and motif pairs in stem and DE cells. Hierarchical clustering of regression weights followed by motif enrichment recovers clusters representing cell type–specific transcription factor DNA-binding motifs. (*F*) Example of individual motifs (*left*, *middle*) that alone do not result in differentially open chromatin but result in differentially open chromatin ESCs in combination (*right*). (*Top* row) Distribution of MIAA-measured accessibility in ESCs and DE cells for KMAC- or DeepAccess-generated motif, tested over 24 neutral sequence backgrounds and randomly shuffled DNA controls (CTRL). (*Bottom* row) Measurements for a particular DeepAccess or KMAC motif, in which each dot represents a single neutral background. The *y*-axis is the difference between endoderm and ESC accessibility, and the *x*-axis is the difference between each DNA sequence and its shuffled control in ESCs.

We noticed previously that the positive control DNA sequences from the Hashimoto et al. (2016) library had higher GC-content than the negative control DNA sequences. To clarify the role of GC-content in driving accessibility, we selected a total of 200 positive and negative control DNA sequences from the Hashimoto et al. (2016) library, which were designed to include a string of motifs that were predicted by a model trained on DNase-seq to have a positive or neutral impact on accessibility (Hashimoto et al. 2016). We selected positive and negative controls with either high GC-content (60%–70%) or low GC-content (30%–50%). We found that in both cell types, positive control DNA sequences drove uniformly and equivalently high accessibility regardless of GC-content (Fig. 3A), suggesting that motifs associated with accessible regions can increase accessibility independently of GC-content. However, in endoderm, positive control DNA sequences for both GC-content bins had increased accessibility compared with negative control DNA sequences with matched GC-content (*P* < 0.001 by Wilcoxon rank-sum test), whereas in ESCs, only the low GC-content bin had differential accessibility between negative and positive controls (*P* < 0.001 by Wilcoxon rank-sum test) (Fig. 3A) because of high accessibility among high-GC neutral DNA sequences. GC-content was positively correlated with accessibility in both ESCs and DE cells among both sets of control DNA sequences (ESC Pearson's *r* = 0.476; DE Pearson's *r* = 0.357), suggesting that GC-content is a contributor to MIAA-measured accessibility alongside motif composition. DeepAccess-predicted accessibility was consistent with MIAA, indicating these effects were to be expected from observations on DNase-seq (Supplemental Fig. S10).

Because this result could be an effect of sequence motifs included in the high-GC-content negative control DNA sequences, we then examined the nucleotide-shuffled DNA sequences that we designed to act as controls for motif activity to see if the effect of GC-content on MIAA accessibility held in random DNA. We found that the GC-content of randomly shuffled sequences correlated with MIAA accessibility in both cell types (Fig. 3B). We also found that accessibility was significantly higher (*P* < 0.001 by one-tailed Wilcoxon signed-rank test) in ESCs compared with endoderm cells across all GC-content bins, except in DNA sequences with <35% GC-content (*N* = 372). Altogether, these results indicate that GC-content alone is a sufficient DNA signal to drive accessibility in both ESCs and endoderm as measured by MIAA and also to drive accessibility differences between these two cell contexts through its heightened impact in ESCs.

Consistent with previous research that suggests a relationship between GC-rich regions and accessibility (Parker et al. 2008; Wang et al. 2012; Schwartz et al. 2019), we found that the top 5000 DE cell–specific regions and the top 5000 ESC-specific regions from DNase-seq have higher GC-content than randomly sampled DNase-inaccessible regions (Supplemental Fig. S10).

We then set out to examine the impact each motif or motif pair sequence derived from our DeepAccess- and KMAC-derived hypotheses beyond the confounding effects of GC-content. We trained a linear regression model to predict MIAA Dpn ratios from GC-content, experimental replicate, and cell type–specific effects for all DNA sequences containing differential motifs or motif pairs. This linear model had good performance on training (Pearson's *r* = 0.6335) and held-out test data (Pearson's *r* = 0.5841) (Fig. 3C; for details, see Supplemental Methods) and significantly improved from regression models that did not include motif effects (adjusted R-squared motif model = 0.398; adjusted R-squared no motif model = 0.095) (Fig. 3D), reinforcing the salient effects of transcription factor binding motifs in controlling accessibility.

We next sought to determine which transcription factor binding motifs most strongly drove differential accessibility between ESCs and endoderm. Because KMAC and DeepAccess identified sequence motifs and motif pairs that could represent the same transcription factor binding site, we clustered the regression weights to identify clusters of motifs and motif pairs representing similar influences on MIAA-measured accessibility (Fig. 3E). We then ran motif discovery on the designed DNA sequences in each cluster to obtain transcription factor candidates (for details, see Supplemental Methods). We identified motifs for known transcription factors such as Pou and Sox motifs as ESC-enriched and motifs for T-box and Fox factors as enriched in DE cells. The regression weights for these differential accessibility–driving motifs were robust, showing high consistency between models trained on biological replicates (Pearson's *r* = 0.963) (Supplemental Fig. S11), indicating that although MIAA correlation at the level of individual DNA sequences is modest, our estimation of motif-level effects is highly reproducible. We also identified motif pair sequences that show interesting nonlinear activity with respect to differential accessibility compared to their motif sequence effects alone (Fig. 3F). In sum, MIAA data enable de novo discovery of features such as GC-content and transcription factor motifs that govern differential chromatin accessibility and validate predictions of motifs impacting differential chromatin accessibility made by DeepAccess.

### Overexpression of DE transcription factors *T* and *Foxa2* increase accessibility of DNA sequences with their DNA-binding motifs

**Figure 4.**
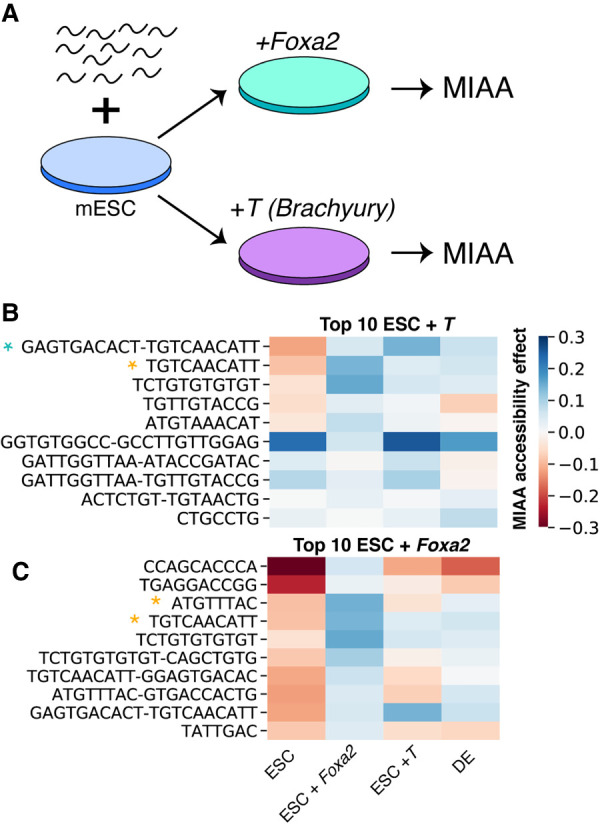
Overexpression of DE lineage-defining transcription factors results in changes to certain motifs representing DNA binding. (*A*) Synthetic DNA sequence library is integrated into ESCs, and *Foxa2* and *T* are overexpressed. (*B*) Regression weight heatmap of top motifs and motif pairs that increase accessibility under *T* overexpression compared with ESCs. Blue star indicates motif visually matches T homodimer in ± orientation that is enriched in ChIP-seq peaks. Yellow star indicates motif is statistically enriched in ChIP-seq peaks of T binding in mouse definitive endoderm cells (*P* < 0.05 HOMER motif enrichment with Benjamini–Hochberg correction). (*C*) Regression weight heatmap of top motifs and motif pairs that increase accessibility under *Foxa2* overexpression compared to ESCs. Star indicates motif is statistically enriched in FOXA2 ChIP-seq peaks in mouse DE cells (*P* < 0.05 HOMER motif enrichment with Benjamini–Hochberg correction).

We then hypothesized we could connect our discovered motifs to transcription factors driving differential accessibility by ectopically expressing transcription factors known to bind to certain enriched motifs. We overexpressed the transcription factors *T* or *Foxa2* in ESCs and measured the accessibility of our DNA sequence library with MIAA (Fig. 4A). We trained a joint regression model to predict condition-specific accessibility with data from four conditions: ESCs, DE cells, ESCs with *Foxa2* overexpression, and ESCs with *T* overexpression (Supplemental Fig. S12; for details, see Supplemental Methods). We then selected the motifs with the greatest positive difference in regression weights between the overexpressed *T* (ESC + *T*) and the ESC conditions. We found that *T* overexpression increases MIAA accessibility most strongly in DNA sequences with a motif pair that partially matches the motif of a T homodimer with two motifs in a minus/plus orientation and is significantly enriched over other dimer orientations in T ChIP-seq peaks (*P* < 0.001 by χ^2^ test) (Supplemental Fig. S13). The second strongest motif is also significantly enriched in T binding in mouse DE as measured by ChIP-seq (*P* < 0.05 under Benjamini–Hochberg multiple hypothesis correction) (Fig. 4B). Overall, only 6/76 motifs or motif pairs showed a significant increase in ESC accessibility upon *T* overexpression (for details, see Supplemental Methods), supporting that T binding is capable of increasing accessibility specifically at motif-containing DNA sequences in a fixed chromatin context.

Similarly, we examined the motifs with the greatest increase in accessibility upon *Foxa2* overexpression and found that the third and fourth top motifs were enriched in sequences from FOXA2 ChIP-seq peaks (*P* < 0.05 under Benjamini–Hochberg multiple hypothesis correction) (Fig. 4C). *Foxa2* overexpression results in more substantial changes in ESC motif accessibility profiles than *T* overexpression (Supplemental Fig. S14), which is consistent with data showing that *Foxa2* overexpression also results in more changes to gene expression (Supplemental Fig. S15), and therefore may lead to secondary chromatin accessibility changes unrelated to the FOXA2 motif. Both *T* and *Foxa2* overexpression resulted in increased accessibility at a TGTCAACATT motif, which is likely because it contains sequences capable of binding both factors and is consequently enriched in both T and FOXA2 ChIP-seq. We also found that both *Foxa2* and *T* overexpression resulted in chromatin accessibility changes that brought cells closer to the MIAA profile of DE cells (Supplemental Fig. S14). Thus, overexpression of individual transcription factors is capable of increasing the chromatin accessibility of a specific cohort of motif-containing sequences in a controlled chromatin context, providing evidence that binding of these factors leads to increased chromatin accessibility.

### Exploration of ordering of ESC and endoderm key transcription factors uncovers subtle TF–TF interactions

**Figure 5.**
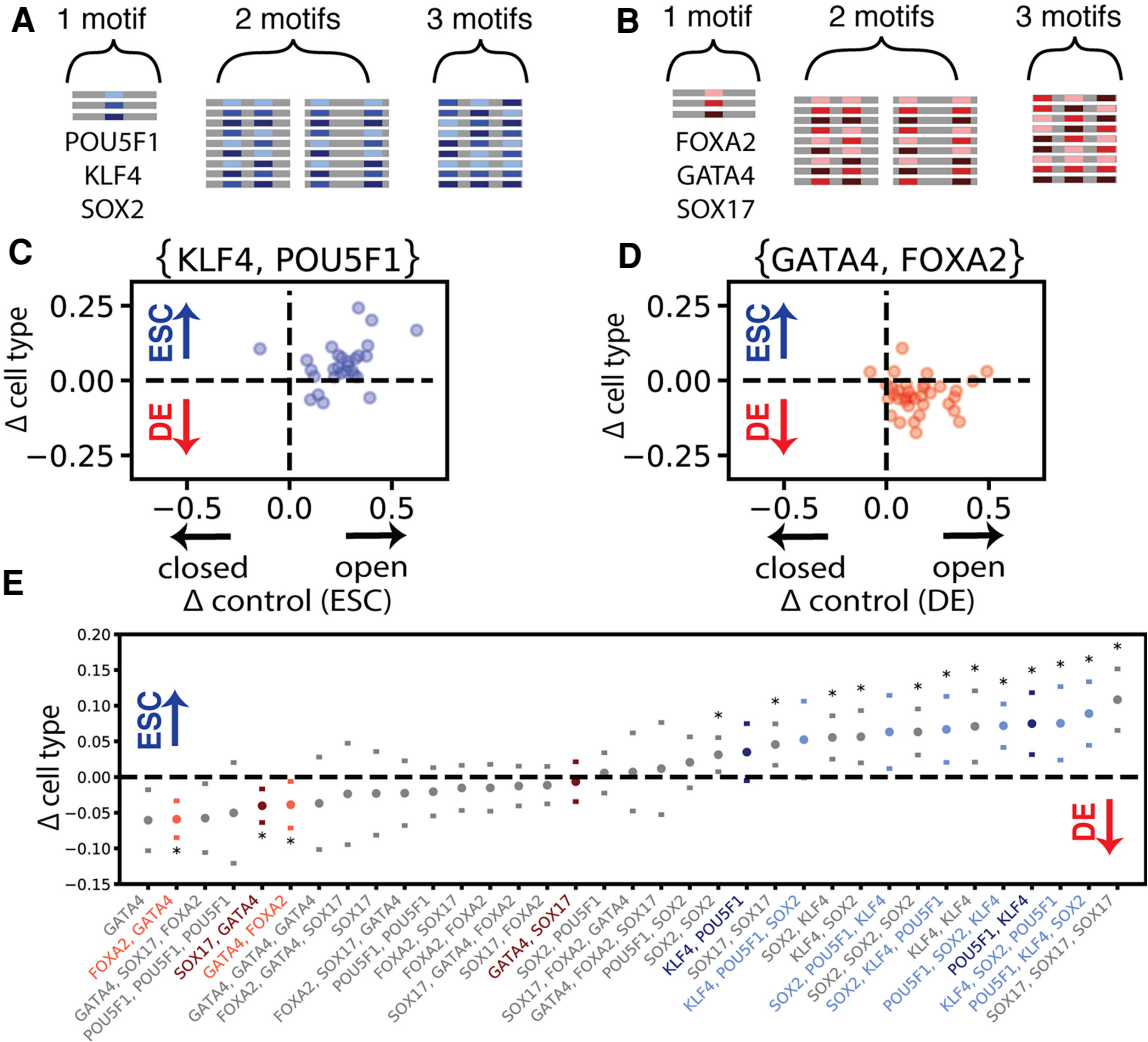
Lineage transcription factor motifs impact chromatin accessibility with preferential spatial ordering. (*A*) DNA sequence construction from the ESC key transcription factors POU5F1, SOX2, and KLF4. (*B*) DNA sequence construction from the DE key transcription factors GATA4, SOX17, and FOXA2. (*C*) Each dot represents a single neutral DNA background sequence that contains one instance of a POU5F1 motif and one instance of a KLF4 motif (two total motif instances per DNA sequence). On the *y*-axis is the difference between endoderm and ESC accessibility, and on the *x*-axis is the difference between each DNA sequence and its shuffled control in ESCs. (*D*) Each dot represents a single neutral DNA background sequence that contains one instance of a GATA4 motif and one instance of a FOXA2 motif (two total motif instances per DNA sequence). On the *y*-axis is the difference between endoderm and ESC accessibility, and on the *x*-axis is the difference between each DNA sequence and its shuffled control in DE cells. (*E*) All motif orderings that had significant accessibility relative to random shuffled DNA controls, ranked by mean differential accessibility. Transcription factor pairs with significant changes in accessibility owing to transcription factor order are colored. Transcription factor orders with significant differential accessibility between DE cells and ESCs are starred (significance computed by paired *t*-test and Wilcoxon signed-rank with Benjamini–Hochberg correction at FDR < 0.05).

Finally, we used MIAA to explore interactions between motifs that are difficult to measure from observational approaches such as DNase-seq because of the lack of suitably controlled genomic motif arrangements. To probe interaction effects over a constrained set of known transcription factors, we designed a new library from the consensus binding motifs of the ESC lineage-defining transcription factors POU5F1, SOX2, and KLF4 (Fig. 5A) and the DE transcription factors FOXA2, SOX17, and GATA4 (Fig. 5B). We tested homotypic DNA sequences with one, two, or three instances of a motif and heterotypic DNA sequences with combinations of motifs with every possible motif ordering (in a single orientation).

We found that single motif instances were able to significantly increase accessibility compared with shuffled DNA sequences for 2/6 transcription factors (SOX17 and GATA4) but were rarely able to make DNA significantly differentially accessible (Supplemental Fig. S16). We note that the consensus motifs for SOX17 and SOX2 are highly similar, sharing a common sequence (CATTGTTT), so it is likely that both Sox factors and possibly others bind to both motifs tested. In contrast, in our DNA sequences containing two motif instances, 17/18 significantly increased accessibility compared with shuffled DNA sequences in at least one cell type (Supplemental Fig. S17), indicating that MIAA is capable of reliably detecting accessibility changes resulting from a minimum of two motif instances and that all six motifs open chromatin in at least one cell type. We then tested for differential accessibility with 6-nt versus 20-nt distance between motifs, which we selected based on literature supporting preferential distances between SOX2 and POU5F1 and between KLF4 and POU5F1 (Guo et al. 2012), and we found that none were significantly sensitive to spacing under multiple hypothesis correction. We found that overall the measured accessibility impact of these motifs did not match well with the expression of the canonical transcription factors that are expected to bind these motifs, suggesting that the MIAA assay measures more than the relative expression of specific transcription factors (Supplemental Fig. S18).

We then examined all homotypic and heterotypic conformations with one, two, or three motif instances for induction of accessibility and differential accessibility. Overall, we found that 35/42 conformations significantly increased accessibility compared with shuffled versions in at least one cell type, and 15 out of 42 motif conformations were statistically significant for differential accessibility induction after multiple hypothesis correction (Fig. 5E). Of these 15 conformations inducing differential accessibility, 10 are heterotypic, with POU5F1-KLF4 combinations and POU5F1-KLF4-SOX2 combinations preferentially driving accessibility in ESCs (Fig. 5C,E; Supplemental Fig. S19) and FOXA2-GATA4 and SOX17-GATA4 combinations driving endoderm accessibility (Fig. 5D,E; Supplemental Fig. S19).

In several cases, homotypic motif arrays showed accessibility patterns inconsistent with the expression of their expected transcription factors. For example, homotypic SOX17 motifs drive ESC-enriched accessibility, and homotypic FOXA2 motifs drive accessibility equivalently in ESCs and endoderm in contrast to the endoderm-specific expression of both transcription factors. Though we chose canonical motifs for factors well known in the literature to be associated with ESCs and endoderm, motifs are often shared by multiple members of a transcription factor family. In fact, it has been shown that FOXD3 binds in ESCs to motifs that will eventually become occupied by FOXA2 in endoderm (Xu et al. 2009). This same effect likely holds for SOX2 and SOX17 as well given the similarity of their motifs.

In addition, we observed several instances of heterotypic combinations of transcription factor motifs in which order (whether a transcription factor motif was closer to the 5′ or 3′ end of the designed ssDNA sequence) had an impact on accessibility. For ESC factor binding motifs, we found that one ordering of POU5F1 and KLF4 more strongly differentially opens chromatin, whereas the other opens chromatin equivalently in both cell types (Fig. 5E). We also found four out of six sequences that contained all three ESC reprogramming motifs were differentially accessible, and the order of these motifs had an impact on the level of differential accessibility (Fig. 5E).

Among endoderm factor motif combinations, we found that particular FOXA2 and GATA4 and SOX17 and GATA4 (Fig. 5E) orientations promoted more differential accessibility. Previous studies have implicated GATA4 and FOXA2 as accessibility-enhancing transcription factors (Cirillo et al. 2002; Sherwood et al. 2014) and have shown that their interaction can drive accessibility changes during endoderm differentiation (Cernilogar et al. 2019). The motif arrangements that produced the most differential MIAA accessibility were also most often enriched in the genome (Supplemental Fig. S20). Because such native genomic instances are rare and confounded by other differences, MIAA provides a more controlled approach to identifying motif arrangements with differential activity.

## Discussion

The MIAA is a new assay for measuring changes in chromatin accessibility caused by short DNA sequences integrated into a fixed locus in the genome. Most prior approaches to understanding the control of chromatin accessibility have used correlative approaches that identify genomic DNA sequences that tend to coincide with accessible chromatin in a particular cell type (Heinz et al. 2010; Setty and Leslie 2015; Corces et al. 2016; Gray et al. 2017; Velasco et al. 2017) or leverage natively occurring SNPs to identify “DNase-QTLs” for which the single nucleotide change correlates with a change in chromatin accessibility (Degner et al. 2012; Grubert et al. 2015), revealing motifs whose disruption is enriched in such variants. MIAA enables screening of an arbitrarily large and diverse library of sequences for their impact on chromatin accessibility. The MIAA assay measures the differential accessibility induced by designed oligonucleotide libraries through the preference for RAR-Dam to bind and methylate accessible DNA. MIAA can measure the relative effects on local chromatin accessibility of many sequences in parallel in a fixed genomic context. This has enabled us to identify candidate accessibility modifiers such as transcription factor binding sites and cooperative interactions between such sites. Notably, because MIAA lacks the ability to measure exact nucleosome positions, it is not suitable to identify classically defined pioneer factors that must be shown to bind to nucleosomal DNA and move or evacuate nucleosomes.

We applied MIAA to study the effects of motifs on differential accessibility between ESC and DE cell states using a number of distinct experimental designs. Through the use of native genomic 100-nt DNA sequences transplanted to a fixed locus, we were able to recapitulate the differential accessibility from native DNase-seq (Pearson's *r* = 0.53; *P* < 0.001), which we believe can be partially attributed to the use of DeepAccess to scan for highly differential native sequences that are more likely to be causal for specifying differential chromatin accessibility. Through examination of randomly shuffled control DNA sequences, we identify a distinction between how a set of natively ESC-specific and endoderm-specific sequences achieved differential accessibility. The natively endoderm-accessible sequences opened chromatin more in endoderm than in ESCs and more than their shuffled versions on average, suggesting the presence of binding sites for endoderm-specific accessibility-promoting transcription factors. On an individual level, only a subset of sequences act in this way, suggesting that a 100-nt DNA sequence does not always fully recapitulate the chromatin accessibility status of native regulatory elements, which often span over a kilobase. This may be caused by the absence in MIAA of specific sequence elements outside the 100-nt sequence that either contribute to or interact with the 100-nt sequence in its native locus.

We found a distinct pattern in the natively ESC-accessible sequences. In this cohort of sequences, MIAA accessibility was higher in ESCs than in endoderm as expected; however, there was no difference between the ESC accessibility of the DNA sequences and their shuffled counterparts. Instead, the accessibility in endoderm was reduced compared with shuffled controls, suggesting that differential accessibility of these sequences was primarily achieved through binding sites that depress accessibility in endoderm. This result indicates that, for the integration locus used in this work, MIAA is capable of measuring sequence-dependent increases and decreases in accessibility. We found suggestive evidence that E-box binding sites used by epithelial–mesenchymal transition driver transcription factors such as Zeb factors may be responsible for this repression, as such binding sites were found in 98% of the DeepAccess-proposed ESC-enriched native genomic sequences and none of the endoderm-enriched native genomic sequences. Because the native genomic sites were selected by DeepAccess based on predicted optimal differential accessibility modeled from DNase-seq regions, it is striking to have detected such a consistent difference in the mechanism of achieving differential accessibility, and it will be intriguing to explore a larger cohort of cell type–specific sequences to determine which mechanism is more common. It is important to note that DeepAccess results will be specific to the cell types that are being compared, which may also explain why DeepAccess did not strongly identify the key ESC transcription factors. We note that our subsequent exploration of POU5F1, SOX2, and KLF4 motif combinations identified a number of designs that consistently yielded ESC-enriched accessibility compared with scrambled versions, indicating that ESCs are also capable of achieving sequence-specific increases in chromatin accessibility.

To identify causal motifs and transcription factors involved in mediating differential chromatin accessibility, we then focused on exploring DNA sequences containing various combinations of sequence motifs. We show that, independently of binding motifs, higher GC-content increases accessibility. In MIAA, we can confirm this to occur in the absence of transcription factor binding motifs because of our use of shuffled versions of each designed DNA sequence. Although it is formally possible that this GC effect is an artifact of the use of Dam methylase, we show that native genomic accessible regions also show elevated GC-content, and it has been reported that transcription factors and DNase I hypersensitive regions are also enriched in GC-rich regions (Wang et al. 2012).

In spite of its importance, predicting MIAA chromatin accessibility of held-out DNA sequences purely based on GC-content yields poor results, whereas much better results are achieved by accounting for binding motifs. Of the motifs that can be confidently matched to known transcription factor families, our results are consistent with the action of known tissue-specific pioneer factors including SOX2 and POU5F1 in ESCs and GATA4 and FOXA2 in the endoderm (Cirillo et al. 2002; Iwafuchi-Doi and Zaret 2014; Soufi et al. 2015). We confirm the role of FOXA2 and T in endoderm-specific chromatin opening by showing that overexpression of these DE transcription factors in ESCs can increase MIAA-measured accessibility significantly in DNA sequences with DNA-binding motifs recognized by these factors. We found that our method of aggregating motif measurements over multiple sequence backgrounds resulted in highly reproducible estimates of motif effects over biological replicates (*r* = 0.963), highlighting the power of MIAA to identify accessibility-altering motifs.

We then designed a library using consensus motifs of several key transcription factors in all possible combinations and orderings, from which we provide evidence that a single binding site is sufficient to increase chromatin accessibility and as few as two binding sites are sufficient to induce differential accessibility between two cell types. These results suggest for the first time that individual transcription factor binding events in the absence of DNA-binding cofactors are capable of altering chromatin accessibility in mammalian cells.

We also found that for motifs known to bind to both ESC and DE transcription factors, motif order has a subtle effect on accessibility, which provides support for specific transcription factor interactions driving accessibility change. This result illustrates the complexity of differential accessibility induction, which cannot simply be distilled to the presence of consensus motifs for differentially expressed transcription factors. In addition to the reuse of genomic motifs by different members of the same transcription factor family in different cell states (Xu et al. 2009), certain transcription factors such as those in the Sox and Pou family can show profoundly distinct binding to specific dimeric motifs that differ in subtle ways (Aksoy et al. 2013). MIAA offers an exciting new way to explore subtleties that influence transcription factor binding logic such as motif ordering, spacing, and dimeric motifs in a controlled genomic setting.

We observed subtle effects of motif order on differential accessibility in our library using consensus motifs of lineage transcription factors, and observed strong changes in accessibility by a motif pair matching a T dimer when *T* was overexpressed, suggesting that MIAA has the capacity to measure the effects of transcription factor interactions on accessibility. Predicting differential accessibility from DNA sequence has been a much more difficult task than predicting cell type–consistent accessibility (Hashimoto et al. 2016; Kelley et al. 2016; Nair et al. 2019), and one possible reason is that more conditional logic is used. The ability of MIAA to obtain sensitive measurements of the effects of specific motif combinations on differential accessibility by exhausting all possible combinations of motifs in a controlled fashion makes MIAA a valuable tool in training accurate predictive models of chromatin accessibility. There are many directions for future work, including a deeper examination of the impact of genomic integration site on local DNA accessibility as well as a further investigation into features such as motif spacing, which are likely to impact transcription factor interaction logic. MIAA may also find an important use in classifying the large collection of SNPs that may impact chromatin accessibility (Degner et al. 2012). Another possible application of MIAA is to understand chromatin accessibility during differentiation by taking measurements at multiple timepoints to discover novel transcription factor regulatory logic, such as switching of binding partners, in developmentally relevant cell types.

## Methods

### DNA sequence library design

All oligonucleotide libraries were ordered from Twist Biosciences. Variable DNA sequences (70–100 nt depending on library) are flanked by 25-nt primer sequences containing a GATC site and homology arms for CRISPR integration. We identified six native genomic sequences of size 100 nt from a pilot experiment that did not drive differential accessibility with MIAA but varied in GC-content. We randomly perturbed these native sequences three times each to obtain a total of 24 neutral sequence backgrounds. For our first experiment, we took each background and inserted either one motif seven times (positions 2, 16, 30, 44, 58, 72, 86) or two motifs in which motif 1 is inserted four times (positions 2, 30, 58, 86) and motif 2 is inserted three times (positions 16, 44, 72). For our second experiment, we limited ourselves to nine backgrounds that we expected to have high reproducibility to the set of 24. In this experiment, we tested sequences of size 70 nt. By using the consensus sequences of known ES key TFs (POU5F1, SOX2, KLF4) or DE key TFs (FOXA2, GATA4, SOX17), we inserted one, two, or three motifs into each sequence. We tested homotypic DNA sequences consisting of one unique motif, as well as heterotypic DNA sequences enumerating all possible motif orders. Consensus motifs for key developmental transcription factors are listed in Supplemental Table S3. Additional hypotheses were tested within MIAA libraries that were not described in this paper. The DNA sequences that were used in this paper are denoted by a column within the Supplemental Data.

### DNA sequence library integration

Electroporations were performed in two to four biological replicates into p2L RAR-DamA126 ESCs (for cell line construction and RARg-DamN126A-V5His construct sequence, see Supplemental Methods). Cells were grown for 5–8 d after electroporation to obtain adequate quantities for doxycycline treatment. When indicated, cells were differentiated to DE before doxycycline treatment.

### High-throughput sequencing

After DpnI/II digestion, fragments are amplified with three steps of PCR. First, PCR primers to sequence outside the homology arms such that only sequences that are properly integrated at the desired locus and that have not been cleaved by the DpnI/II enzyme are amplified (13 cycles). The second PCR step and third PCR steps further amplify sequences and add adaptors for Illumina sequencing. For primer information and further details, see Supplemental Methods. Samples were sequenced on an Illumina NextSeq 550 instrument at the Harvard Medical School Biopolymers Facility or the MIT BioMicro Center.

### DNA sequence library processing

Reads were mapped to library DNA sequences by taking the reverse complement to the raw read, in which the first N nucleotides (between 70 and 100 based on the size of the designed sequence) are the designed variable DNA sequence. Perfect matches were counted using a custom R script (Supplemental Code). Reads were normalized to reads per million over the total number of reads in the digest. DNA sequences were kept if they had a threshold number of total normalized reads over all replicates, based on the observation of high standard deviation at low total read counts. The threshold was selected based on visual inspection and can be found in the Supplemental Code. Once reads were normalized and high variability DNA sequences filtered, MIAA accessibility was computed as a proportion of DpnI/II read counts DpnII/(DpnI + DpnII).

### DeepAccess model and motif importance

We obtain DNase-seq regions using the 100 nt centered at the MACS2 narrow peak call. Accessibility prediction is treated as a multitask classification problem, in which each genomic sequence (100 bp) is associated with a two-dimensional bit vector representing whether the sequence is open in each cell type (ESC and DE cell). We trained an ensemble of 10 convolutional neural networks. For specific details on network architecture, see Supplemental Methods. The fully connected output layer present in all neural network architectures contains two neurons with a sigmoid activation function that returns a value between zero and one, which represents the probability of the predicted DNA “openness” in each of the two cell types. DeepAccess is trained on a balanced data set with 400,000 sequences across four possible classification scenarios of a sequence (1) open in endoderm cells and closed in ESCs, (2) open in ESCs and closed in endoderm cells, (3) open in both cell types, or (4) closed in both cell types. A test set of 22,357 sequences is held out for performance evaluation.

We extracted motifs from DeepAccess by applying smoothed gradient ascent to score each nucleotide in the 100-nt DNA sequence by its importance for predicting the output (Simonyan et al. 2013; Smilkov et al. 2017) and multiplied times the input (a one-hot encoding of the DNA sequence) because gradients will assign nonzero values to DNA characters not present in the sequence. To obtain sequence importance for features that drive accessibility differentially between DE cells and ESCs, we set the gradient loss to the difference between the predicted accessibility of two cell types. We then selected windows of size 10 with the highest ensemble weighted average saliency over a set of 5000 training sequences and used those as the DeepAccess-derived motifs. We also extracted the top motifs with the highest increase in saliency of differential accessibility between the CNN without trainable hidden layers and the CNNs with hidden layers, which represent motifs that gain importance from the CNNs that learn relationships between motifs.

## Data access

All raw and processed sequencing data generated in this study have been submitted to the NCBI Gene Expression Omnibus (GEO; https://www.ncbi.nlm.nih.gov/geo/) under accession number GSE145920. Prefiltered unnormalized MIAA read counts are available as Supplemental Data. Accession numbers for previously published DNase-seq, ChIP-seq, and RNA-seq data that were used in this study are listed in Supplemental Table S2. Code for DeepAccess accessibility prediction and motif extraction is available at GitHub (https://github.com/gifford-lab/DeepAccess) and as Supplemental Code. Code for MIAA library processing and producing manuscript figures is available at GitHub (https://github.com/gifford-lab/MIAA-analysis) and as Supplemental Code.

## Competing interest statement

The authors declare no competing interests.

## Supplementary Material

Supplemental Material
