# Supplementary material for: Identification of determinants of differential chromatin accessibility through a massively parallel genome-integrated reporter assay
Source: Genome Res. 2020 Oct;30(10):1468–80. doi: 10.1101/gr.263228.120 (PMC7605270; doi:10.1101/gr.263228.120)
Supplement: Supplemental Material [file supp_30_10_1468__index.html]

Identification of determinants of differential chromatin accessibility through a massively parallel genome-integrated reporter assay — Supplemental Material 

# Identification of determinants of differential chromatin accessibility through a massively parallel genome-integrated reporter assay

## Supplemental Material

- Supplemental\_Code.zip
- Supplemental\_Material.pdf
- Supplemental\_Methods.docx
- GSE145920\_phrase\_libraries\_raw\_counts.xlsx
