# Supplementary material for: Identification of determinants of differential chromatin accessibility through a massively parallel genome-integrated reporter assay
Source: Genome Res. 2020 Oct;30(10):1468–80. doi: 10.1101/gr.263228.120 (PMC7605270; doi:10.1101/gr.263228.120)
Supplement: Supplemental Material [file supp_gr.263228.120_Supplemental_Material.pdf]

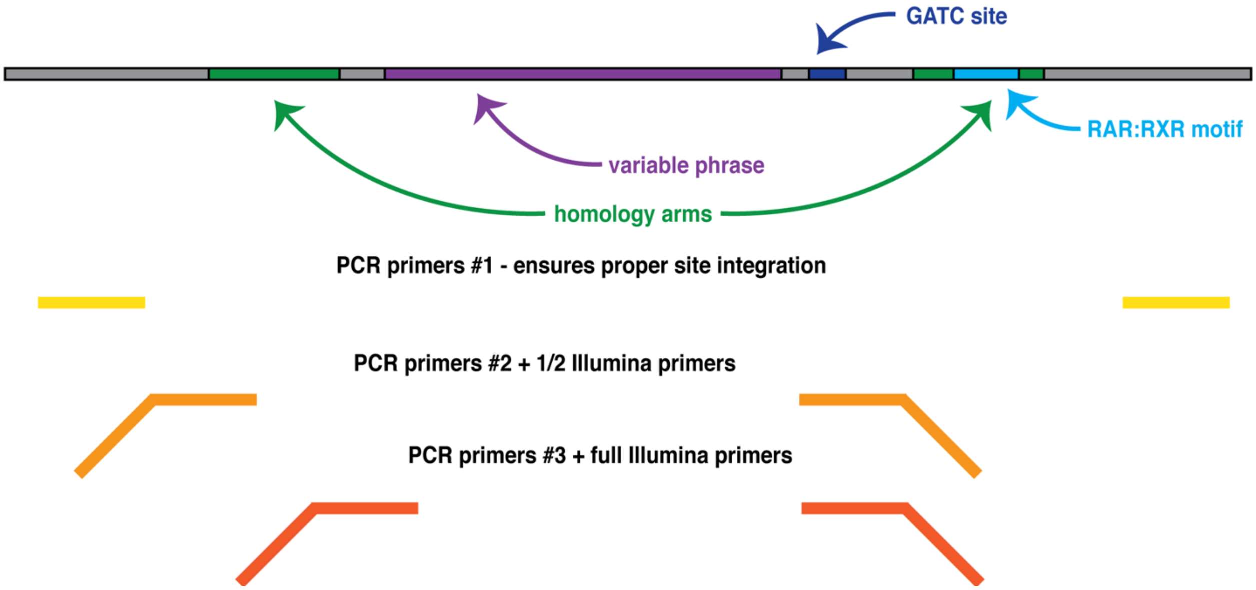

Supplemental Figure S1. MIAA PCR steps to select for proper integration and uncleaved library phrases. First set of PCR primers are designed to select for sequences that have been integrated at the specific genomic locus and are uncleaved by DpnI/II enzyme. Second set and third round of PCR primers enrich for add Illumina PE primers.

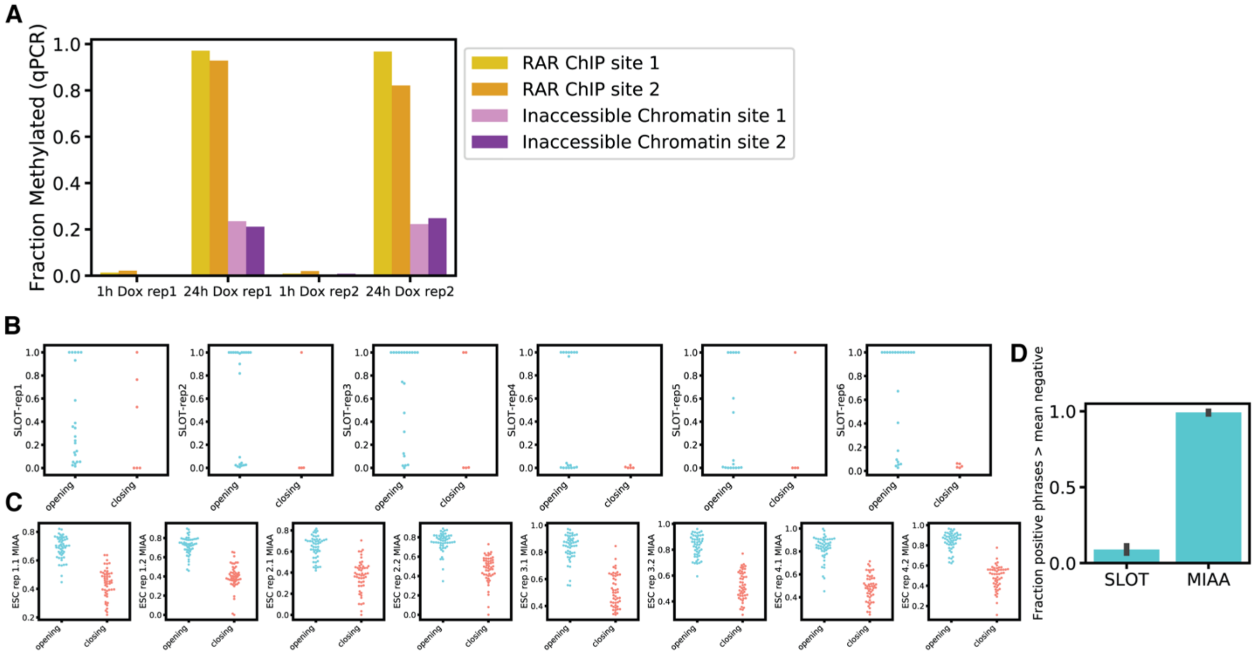

Supplemental Figure S2. RAR-Dam methyltransferase reliably measures accessibility. A) qPCR of fraction of methylation by RAR-Dam in RARG ChIP sequence sites (positive controls) and inaccessible chromatin (negative controls) shows methylation fraction dynamic range from 0%-100%. At 24h after RAR-Dam activation, inaccessible loci have ~20% methylation. B) SLOT measurements of control phrases designed to optimally open chromatin based on predictions from a synergistic chromatin model. C) MIAA measurements of control phrases designed to optimally open chromatin based on predictions from a synergistic chromatin model. D) Overall separation of accessibility measurements by closing phrases and opening phrases reported by fraction of opening category phrases (n=50) more accessible than mean accessibility of closing category phrases (n = 50).

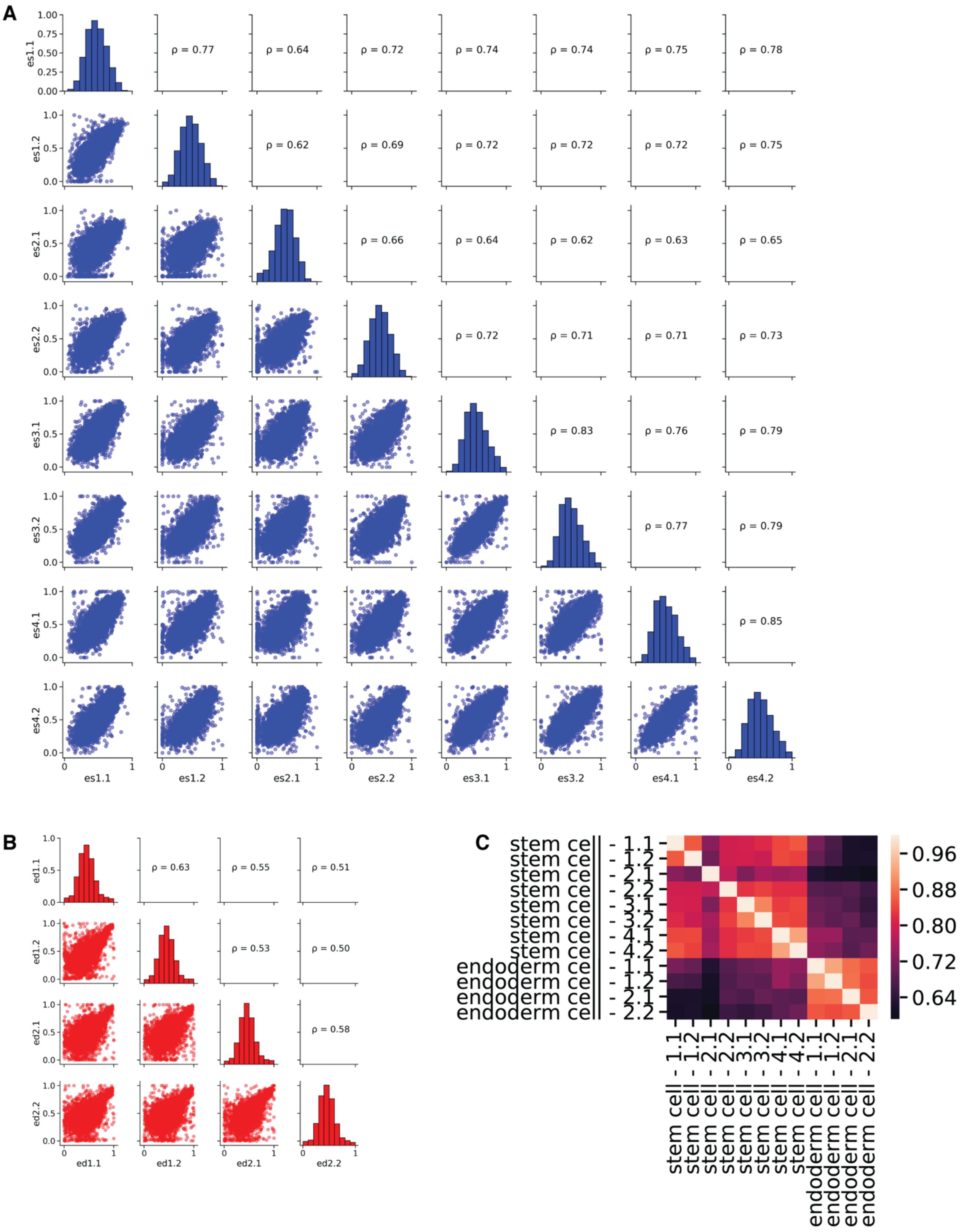
A
es1.1
es1.2
es2.1
es2.2
es3.1
es3.2
es4.1
es4.2
ρ = 0.77
ρ = 0.64
ρ = 0.72
ρ = 0.74
ρ = 0.74
ρ = 0.75
ρ = 0.78
ρ = 0.62
ρ = 0.69
ρ = 0.72
ρ = 0.72
ρ = 0.72
ρ = 0.75
ρ = 0.66
ρ = 0.64
ρ = 0.62
ρ = 0.63
ρ = 0.65
ρ = 0.72
ρ = 0.71
ρ = 0.71
ρ = 0.73
ρ = 0.83
ρ = 0.76
ρ = 0.79
ρ = 0.77
ρ = 0.79
ρ = 0.85
B
ed1.1
ed1.2
ed2.1
ed2.2
ρ = 0.63
ρ = 0.55
ρ = 0.51
ρ = 0.53
ρ = 0.50
ρ = 0.58
C
stem cell - 1.1
stem cell - 1.2
stem cell - 2.1
stem cell - 2.2
stem cell - 3.1
stem cell - 3.2
stem cell - 4.1
stem cell - 4.2
endoderm cell - 1.1
endoderm cell - 1.2
endoderm cell - 2.1
endoderm cell - 2.2
0.96
0.88
0.80
0.72
0.64

Supplemental Figure S3. MIAA experiments are reproducible. A) Dpn proportion of all stem cell replicate experiments over 6K phrase library. Statistic reported (ρ) is Pearson's correlation coefficient. B) Dpn proportion of all endoderm replicate experiments over 6K phrase library. R reported is Pearson correlation coefficient. C) Heatmap of Pearson correlation of Dpn proportion over universally opening, universally closing and background phrases in both cell types.

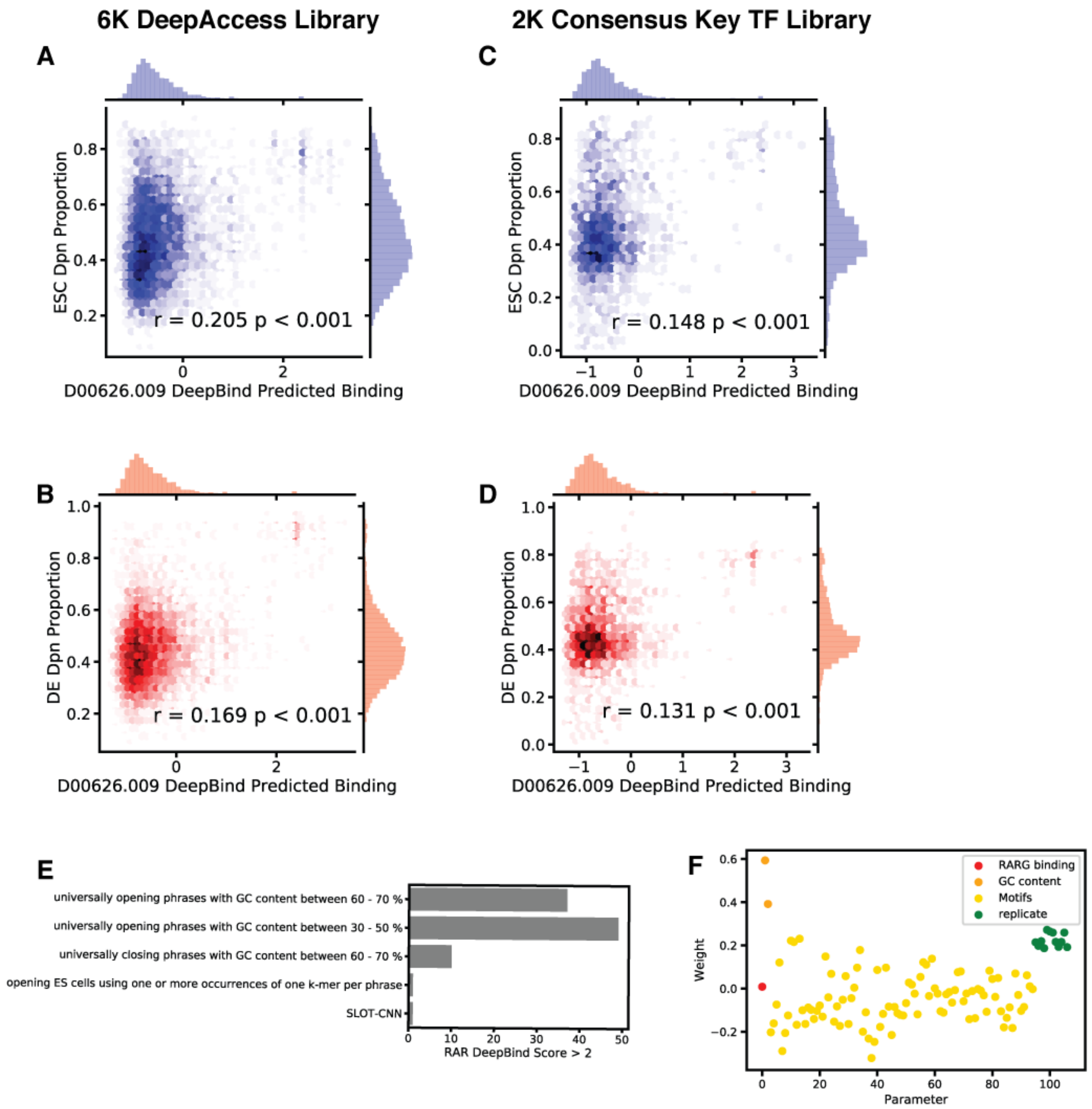

Supplemental Figure S4. Impact of retinoic acid receptor binding on MIAA-measured accessibility. A) DeepBind RXRA ChIP-seq predicted binding to variable phrase compared to MIAA Dpn proportion in stem cells in 6K phrase DeepAccess/KMAC library. B) DeepBind RXRA ChIP-seq predicted binding to variable phrase compared to MIAA Dpn proportion in definitive endoderm in 6K phrase DeepAccess/KMAC library. C) DeepBind RXRA ChIP-seq predicted binding to variable phrase compared to MIAA Dpn proportion in stem cells in 2K phrase consensus transcription factor motif library. D) DeepBind RXRA ChIP-seq predicted binding to variable phrase compared to MIAA Dpn proportion in definitive endoderm in 2K phrase consensus transcription factor motif library. E) Counts of phrases over categories in 6K phrase library that have a DeepBind predicted binding score greater than 2 are all from control sequences. F) Linear regression model trained on 6K library to predict MIAA measured accessibility in stem cells and definitive endoderm predicts very minimal contribution of DeepBind RARG binding score to DeepAccess model.

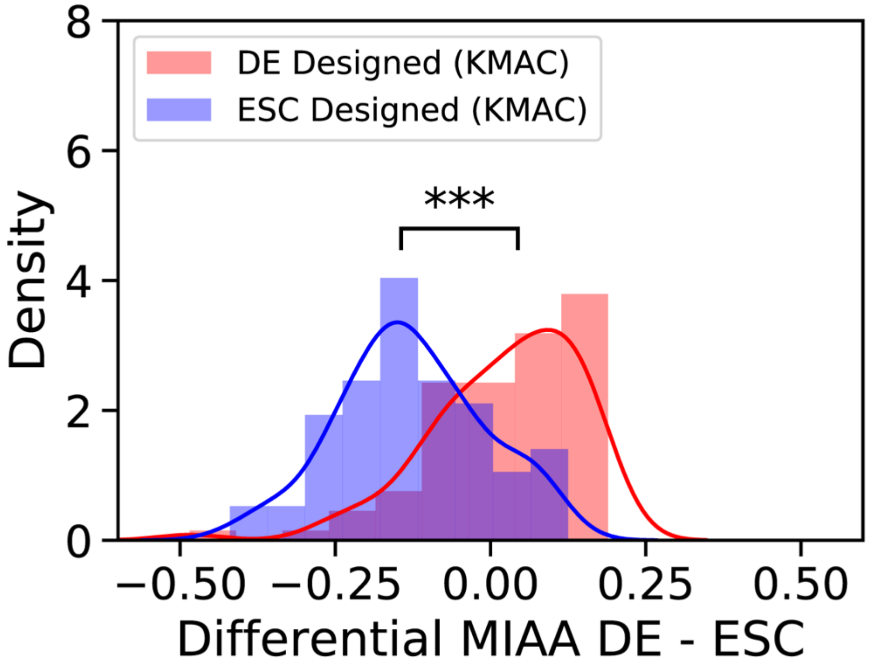

Supplemental Figure S5. DNase-seq based differentially accessible motifs are differentially accessible as measured by MIAA. DNA sequences designed to pack KMAC-derived enriched motifs to be accessible in definitive endoderm (n = 77) or stem cells (n = 87) are differentially accessible ($p < 0.001$ by Wilcoxon Rank Sum Test).

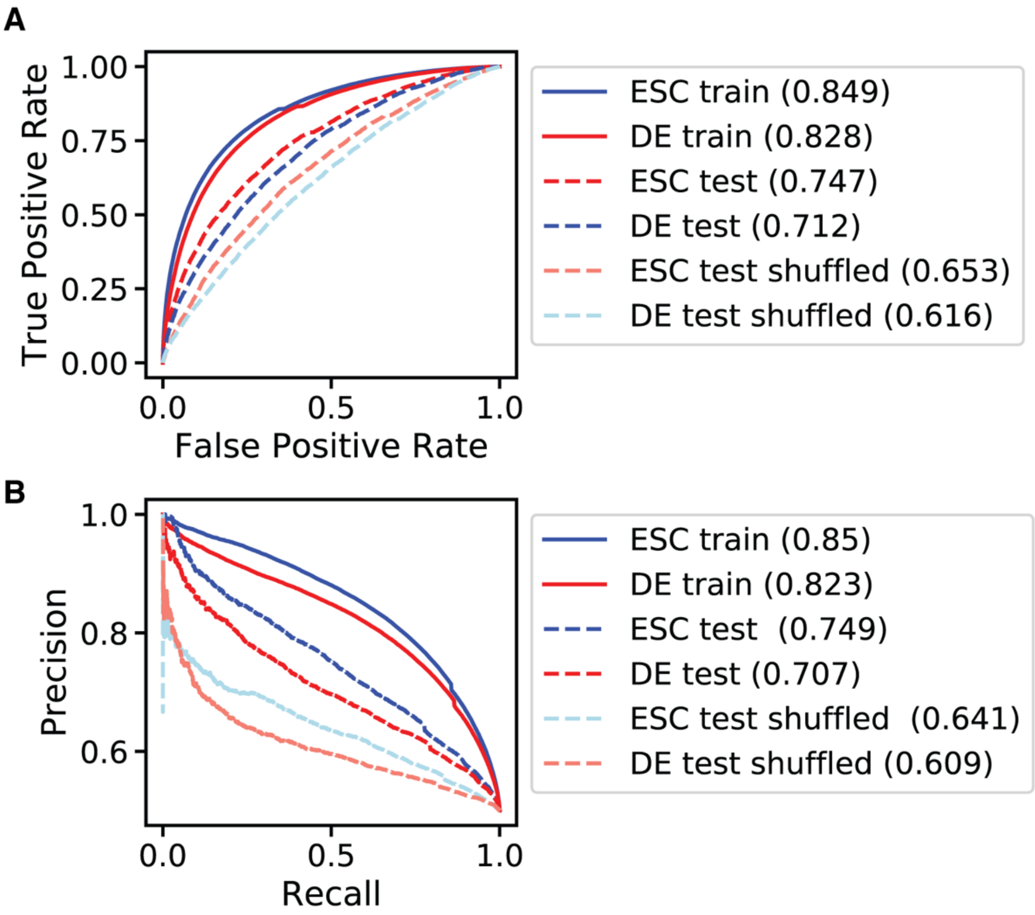

Supplemental Figure S6. DeepAccess accurately predicts chromatin accessibility on training (N=400,000) and held-out test data (N=22,357). A) ROC curves - legend reports AUROC in parenthesis. B) Precision-recall curves – legend reports weighted average precision in parenthesis.

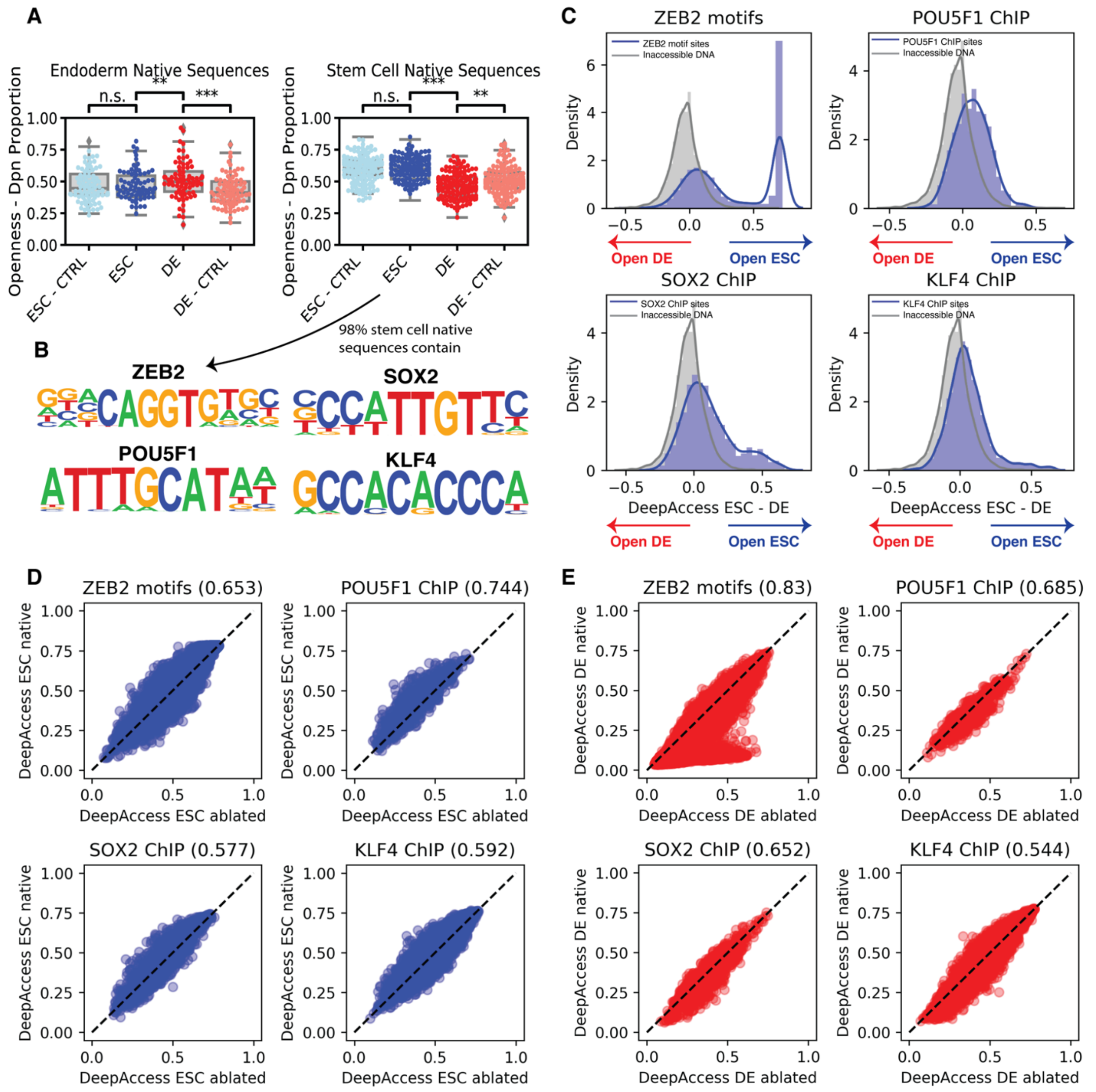

Supplemental Figure S7. DeepAccess predicts effects of key stem cell transcription factor motifs on chromatin accessibility. A) Native genomic sequences relative to randomly shuffled DNA controls selected based on optimal differential accessibility predicted by DeepAccess. For sequences hypothesized to be differentially opening definitive endoderm, sequences are both significantly opening in endoderm relative to stem cell ($p < 0.001$ by paired t-test) and relative to randomly shuffled DNA ($p < 0.001$ by paired t-test). For sequences hypothesized to be differentially opening in stem cells, sequences are significantly opening in stem cell relative to definitive endoderm ($p < 0.001$ by pair t-test) but are not differentially opening relative to random DNA in stem cells. However, sequences were differentially closing relative to random DNA in endoderm ($p < 0.01$ by paired t-test). B) HOMER motifs used for motif enrichment. C) DeepAccess predictions for ZEB motif sites in stem cell accessible DNA, POU5F1 ChIP sites,

SOX2 ChIP sites, and KLF4 ChIP sites show that sites containing motifs are predicted to be more accessible than inaccessible DNA. Strongest predicted effect on differential accessibility is for ZEB motifs. D) Comparison of DeepAccess predictions for stem cell accessibility compared to sequences where motif instance is ablated (random shuffled DNA) show presence of POU5F1 has strongest effect on stem cell accessibility. Fraction of sequences with greater predicted accessibility compared to motif-ablated sequence in parenthesis. E) Comparison of DeepAccess predictions for stem cell accessibility compared to sequences where motif instance is ablated (random shuffled DNA) show presence of ZEB has strongest effect on stem cell accessibility. Fraction of sequences with greater predicted accessibility compared to motif-ablated sequence in parenthesis.

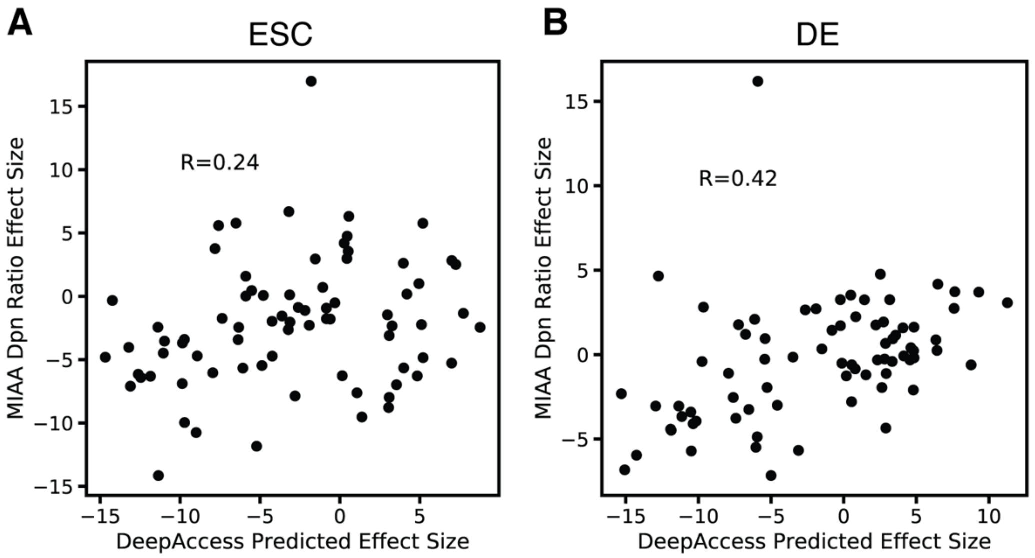

Supplemental Figure S8. DeepAccess predictions of motif effects from homotypic and heterotypic phrases compared to shuffled controls. Each dot is the measured effect size over 24 backgrounds containing either 6 instances of one motif (homotypic) or 3 instances of two motifs (heterotypic). A) Effects of motifs relative to paired randomly shuffled control phrases in mouse embryonic stem cells in MIAA assay compared to predictions from DeepAccess. B) Effects of motifs relative to paired randomly shuffled control phrases in definitive endoderm cells in MIAA assay compared to predictions from DeepAccess. The correlation reported is the Pearson correlation coefficient (r).

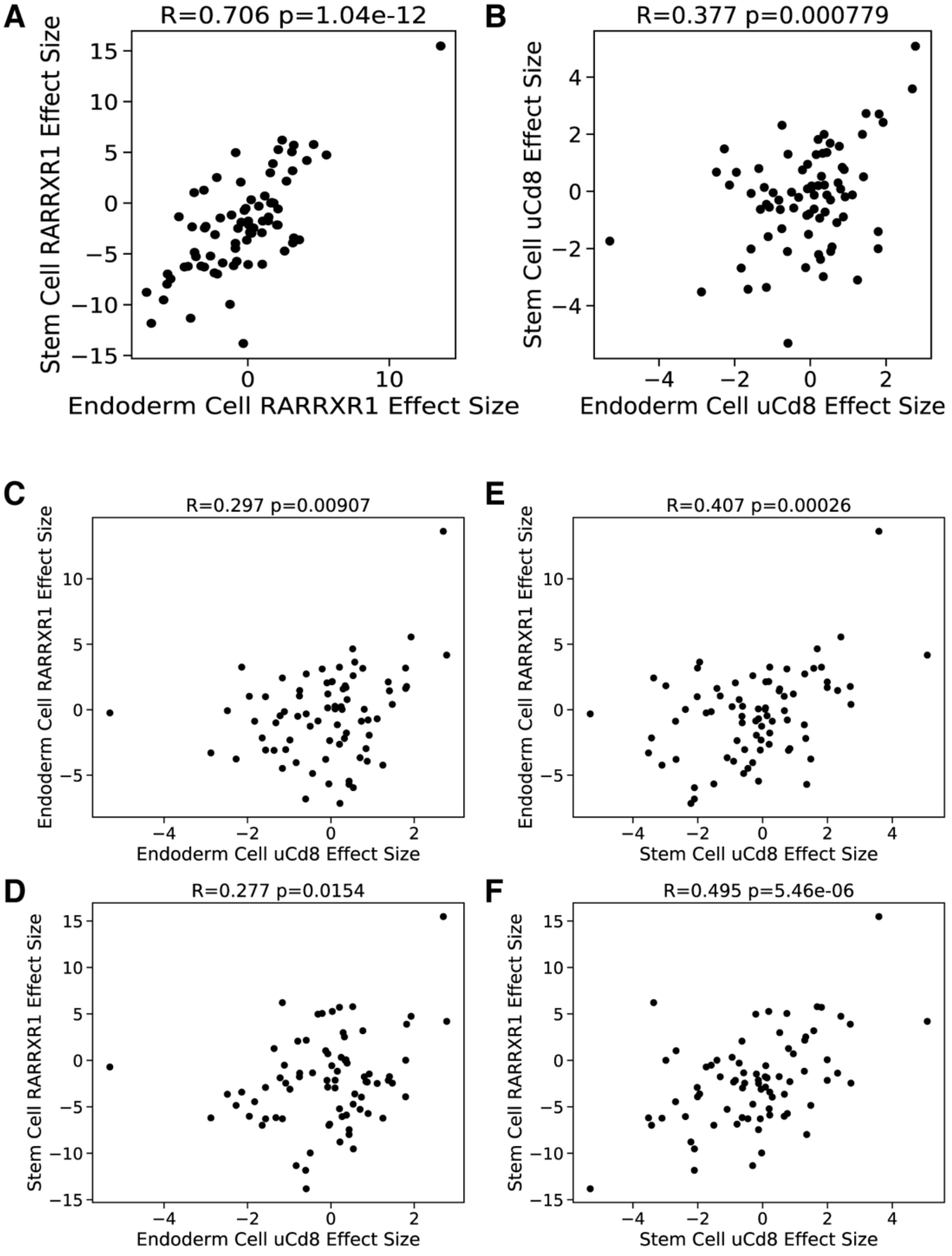

Supplemental Figure S9. MIAA experiments are reproducible in uCd8 genomic integration locus. Each dot represents the effect size by paired t-test for a motif in 24 neutral backgrounds compared to paired shuffled controls. A) Comparison of effect size between cell types in RARXR1 locus shows high correlation. B) Comparison of effect size between cell types in uCd8 locus shows lower correlation, indicating lower bound on significance of the results. C) Comparison between effect size in definitive endoderm in both loci is higher than between stem cell RARRXR1 and endoderm uCd8 (D), but lower than correlation between endoderm RARRXR1 and stem cell uCd8 (E). Effect size between stem cell RARRXR1 and stem cell uCd8 is highest out of all cross-loci comparisons.

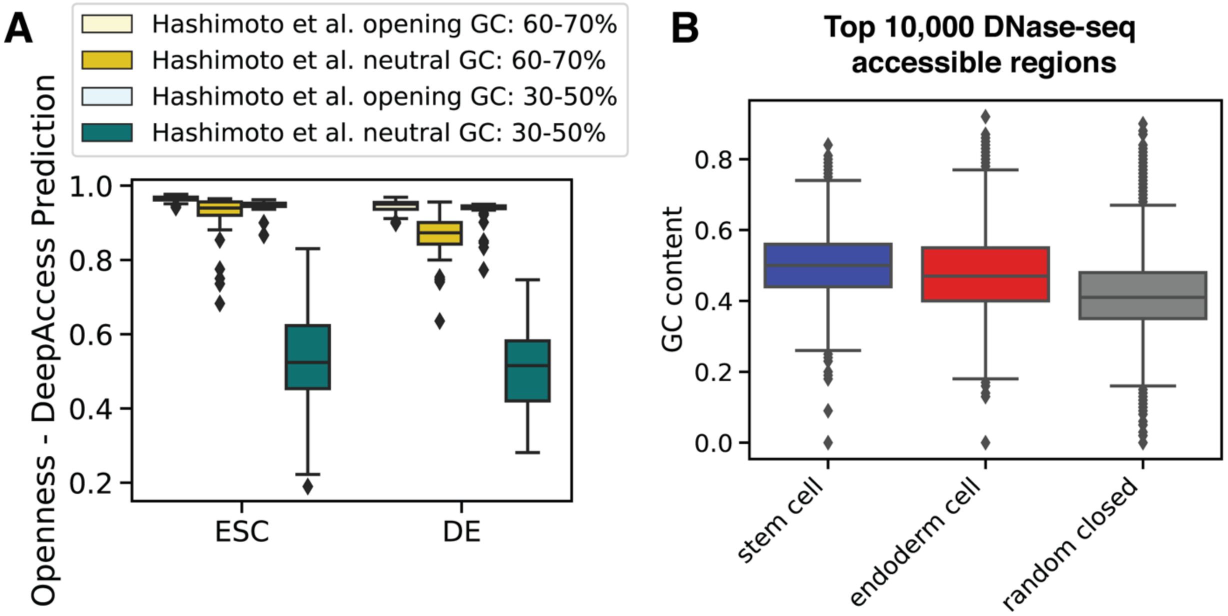

Supplemental Figure S10. A) DeepAccess predicted accessibility of Hashimoto et al. control phrases shows similar patterns of strong effect of GC-content on accessibility as seen with MIAA. B) GC-content of top 5,000 most accessible genomic regions in stem cell and definitive endoderm is higher than in randomly selected closed regions ($p < 0.001$ by rank sum test for stem cell to random and $p < 0.0001$ by rank sum test definitive endoderm to random).

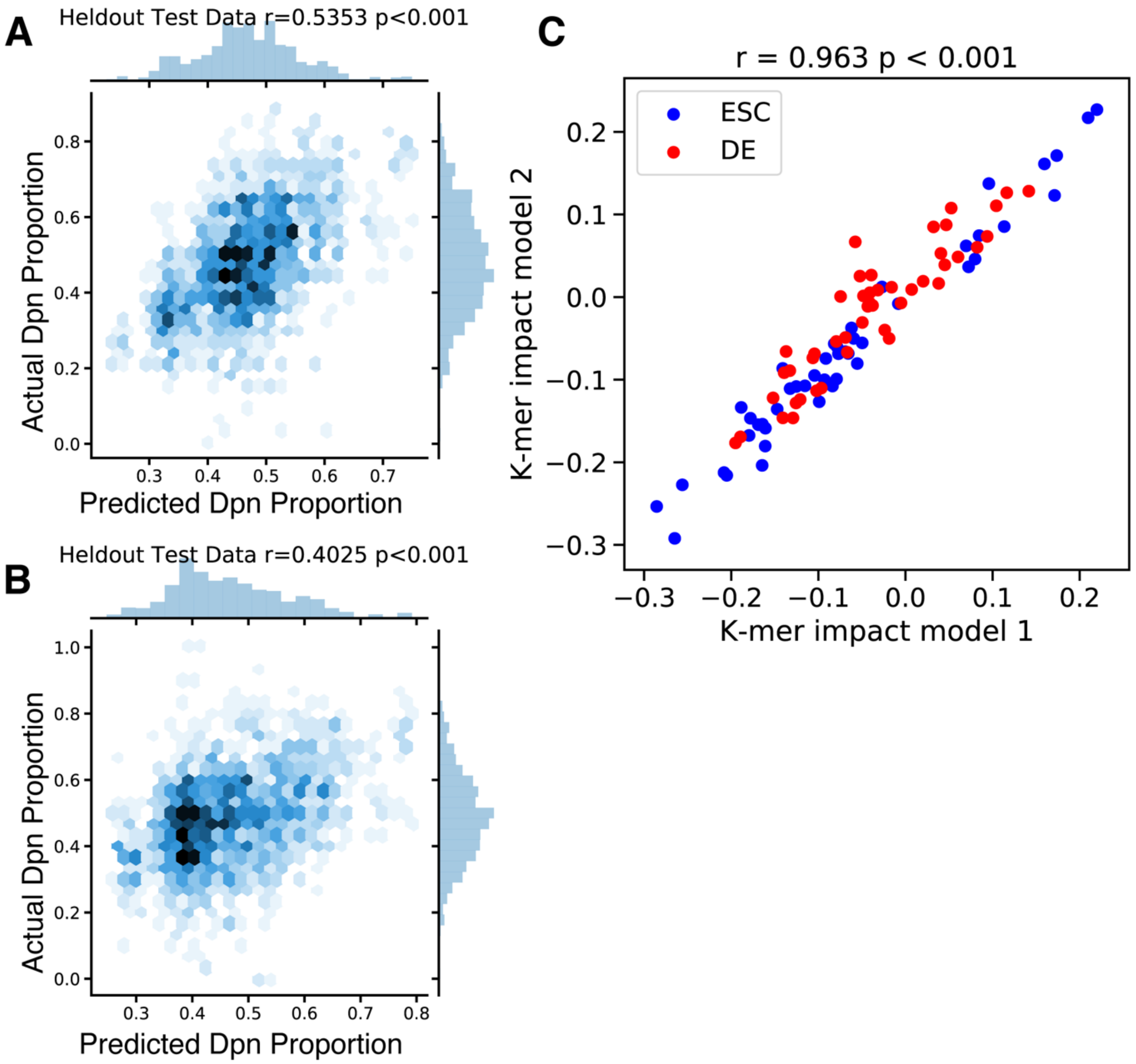

Supplemental Figure S11. Regression model weights reproducible across biological replicates. We trained 2 regression models with features of GC-content, replicate, and cell type specific motifs. Model 1 is trained on experiments (es1.1, es1.2, ed1.1, ed1.2) and Model 2 is trained on experiments (es2.1, es2.2, ed2.1, ed2.2). A) Performance of Model 1 on held out test data. B) Performance of Model 2 on held out test data. C) Estimated cell type-specific motif weights of 2 regression models show high correlation. All reported R values are Pearson correlation coefficients.

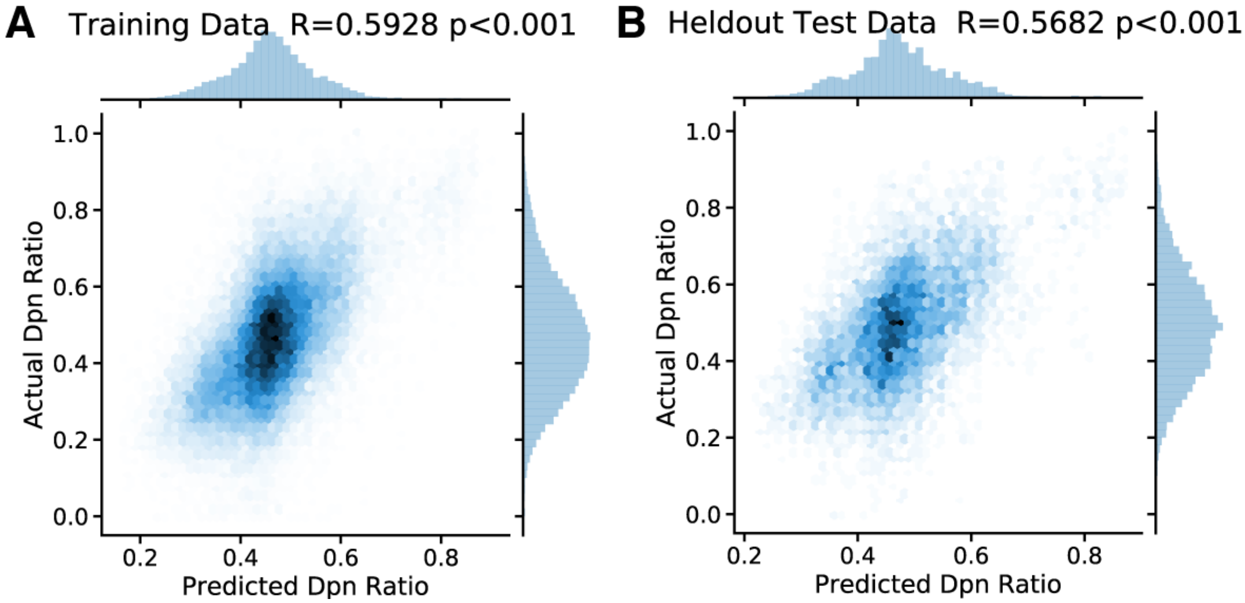

Supplemental Figure S12. Training (n=34,528) and held-out (n=7,280) predictions of model trained on stem cell, definitive endoderm, Brachyury over-expression, and FoxA2 over-expression. The correlation reported is the Pearson correlation coefficient (r).

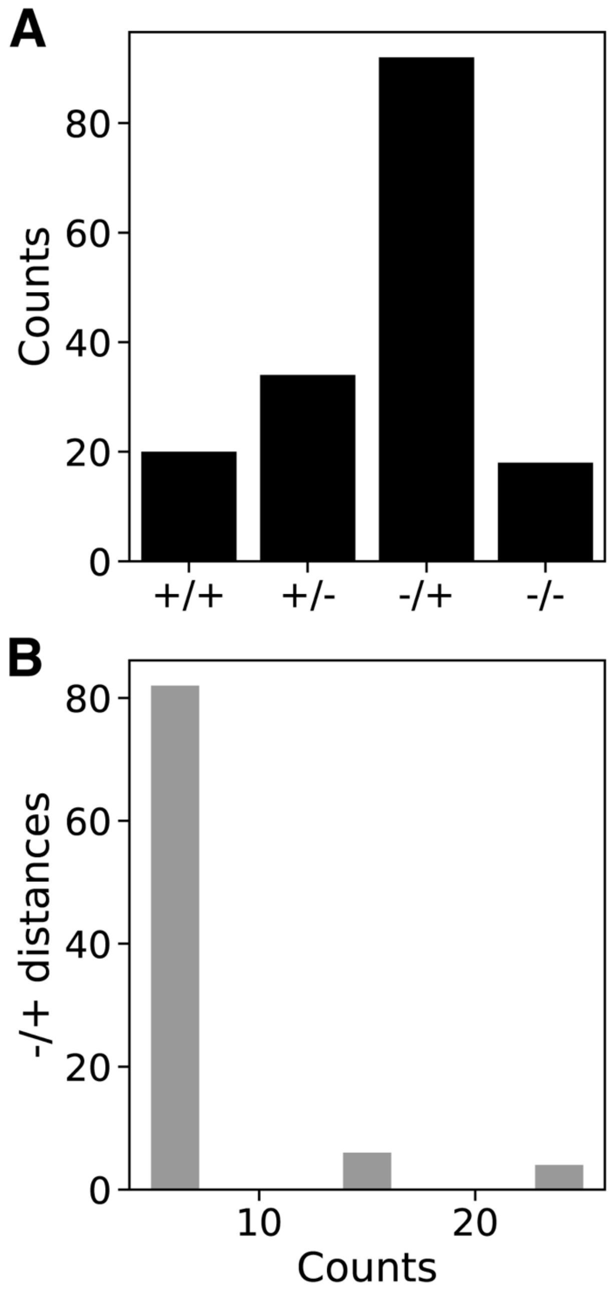

Supplemental Figure S13. T dimers orientation and distance enrichment. A) Counts of T dimer motif orientations in T ChIP-seq peaks show statistical enrichment of motif pairs in '-/+' orientation (p-value < 0.001 by Chi-squared test). B) Majority of '-/+' motif instances (80/92) are 5 bp apart, indicating a spatial preference from dimerization.

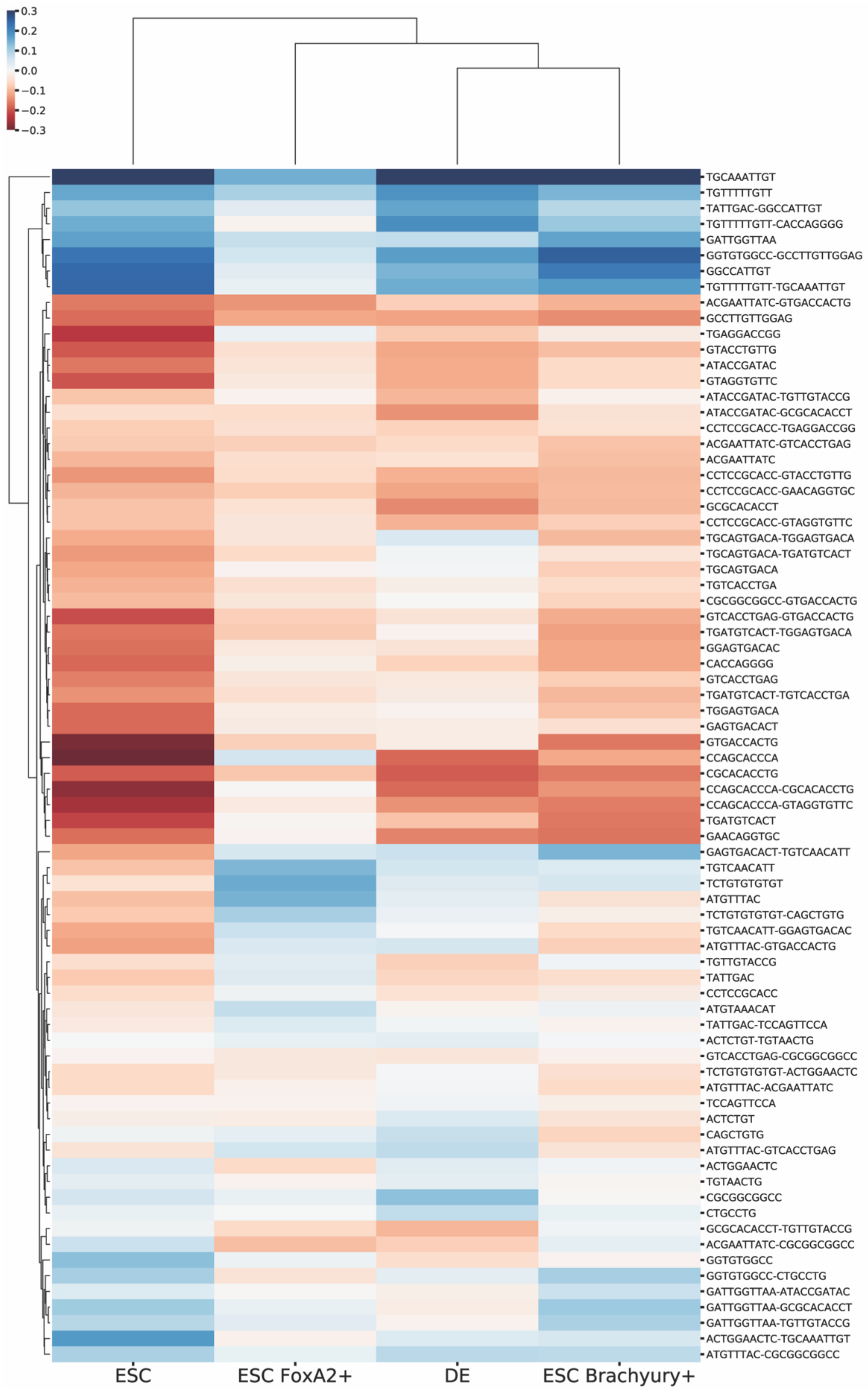

Supplemental Figure S14. Full regression weights for all motifs for stem cells, definitive endoderm, *Foxa2* over-expression, and *T (Brachyury)* over-expression.

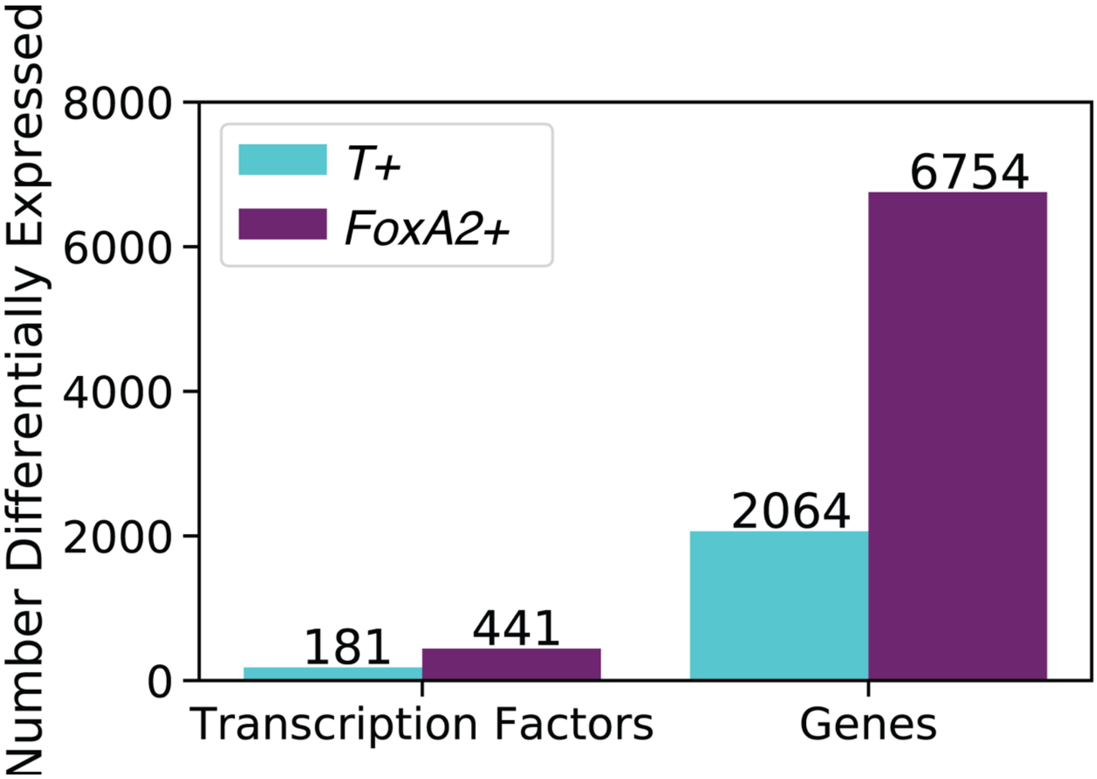

Supplemental Figure S15. Differential expression analysis from over-expression of *T* and *Foxa2* in *T/Eomes* knock-out definitive endoderm cells supports that more transcription changes occur due to *Foxa2* expression.

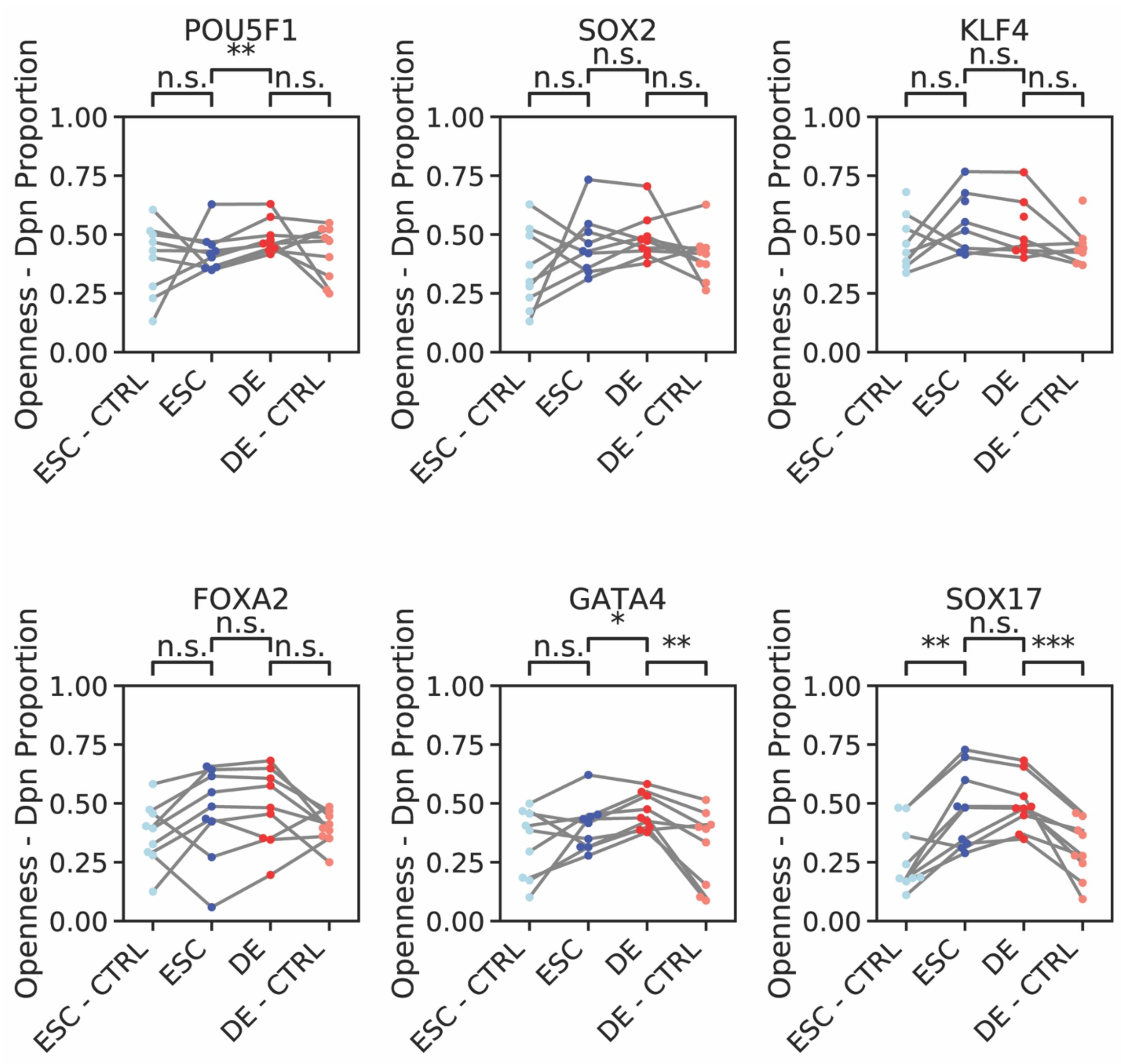

Supplemental Figure S16. MIAA accessibility from single instances of pioneer transcription factor motifs. Single consensus motif instances of pioneer transcription factors inserted into neutral sequence backgrounds generally do not drive differential accessibility except GATA4, which is insignificant when adjusted for multiple hypothesis testing. POU5F1 drives differential accessibility and opening DE, but does not drive differential opening when compared to shuffled controls. All significance scores reported are paired *t*-test (not multiple hypothesis corrected).

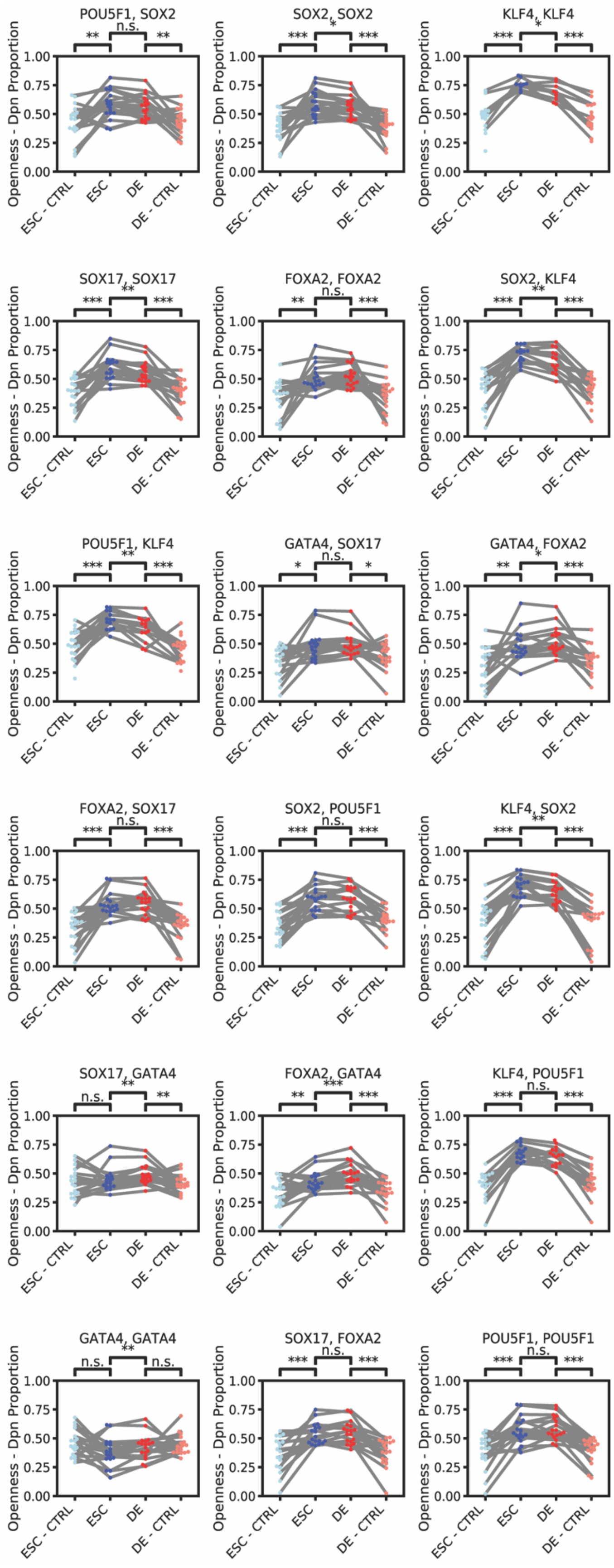

POU5F1, SOX2
** n.s. **
SOX2, SOX2
*** * ***
KLF4, KLF4
*** * ***
SOX17, SOX17
*** ** ***
FOXA2, FOXA2
** n.s. ***
SOX2, KLF4
*** ** ***
POU5F1, KLF4
*** ** ***
GATA4, SOX17
* n.s. *
GATA4, FOXA2
** * ***
FOXA2, SOX17
*** n.s. ***
SOX2, POU5F1
*** n.s. ***
KLF4, SOX2
*** ** ***
SOX17, GATA4
n.s. ** **
FOXA2, GATA4
** *** ***
KLF4, POU5F1
*** n.s. ***
GATA4, GATA4
n.s. ** n.s.
SOX17, FOXA2
*** n.s. ***
POU5F1, POU5F1
*** n.s. ***
Openness - Dpn Proportion
1.00
0.75
0.50
0.25
0.00
ESC - CTRL
ESC
DE
DE - CTRL

Supplemental Figure S17. Phrases containing two motif instances are able to open chromatin in 17/18 cases ($p < 0.05$; Benjamini Hochberg multiple hypothesis correction) and differentially open chromatin between stem cell and endoderm in 9/18 cases ($p < 0.05$; Benjamini Hochberg multiple hypothesis correction). All significance values reported are paired *t*-test (not multiple hypothesis corrected).

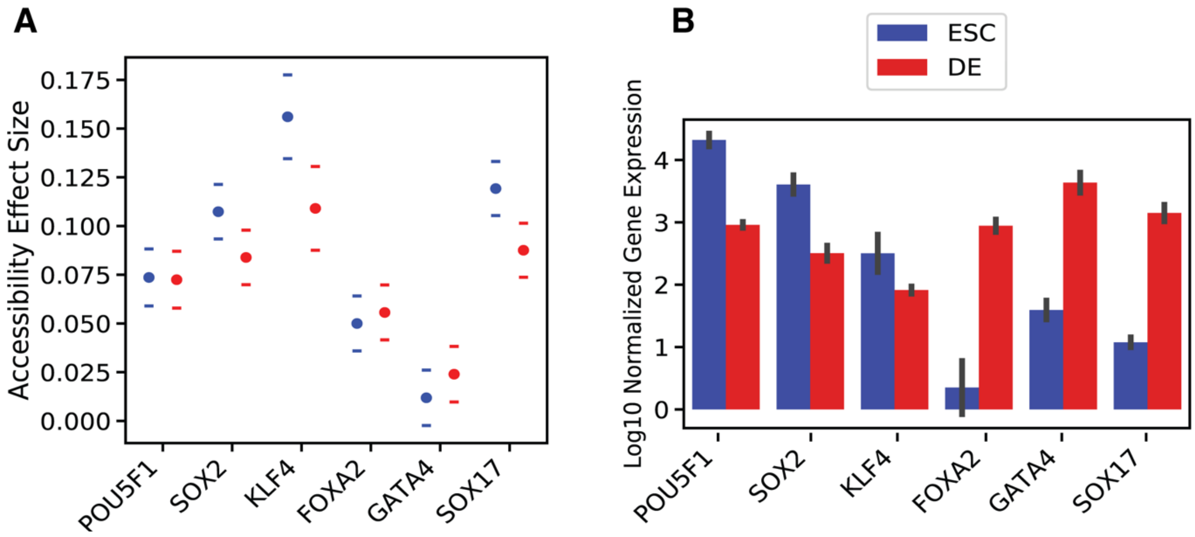

Supplemental Figure S18. MIAA-measured accessibility shows impacts beyond transcription factor expression. A) Effect of transcription factor sequence motifs on cell type-specific accessibility estimated by regression model incorporating GC-content and replicate effects (mean and 95% confidence intervals). B) RSEM normalized gene expression as measured by RNA-seq for transcription factors in stem cell and definitive endoderm.

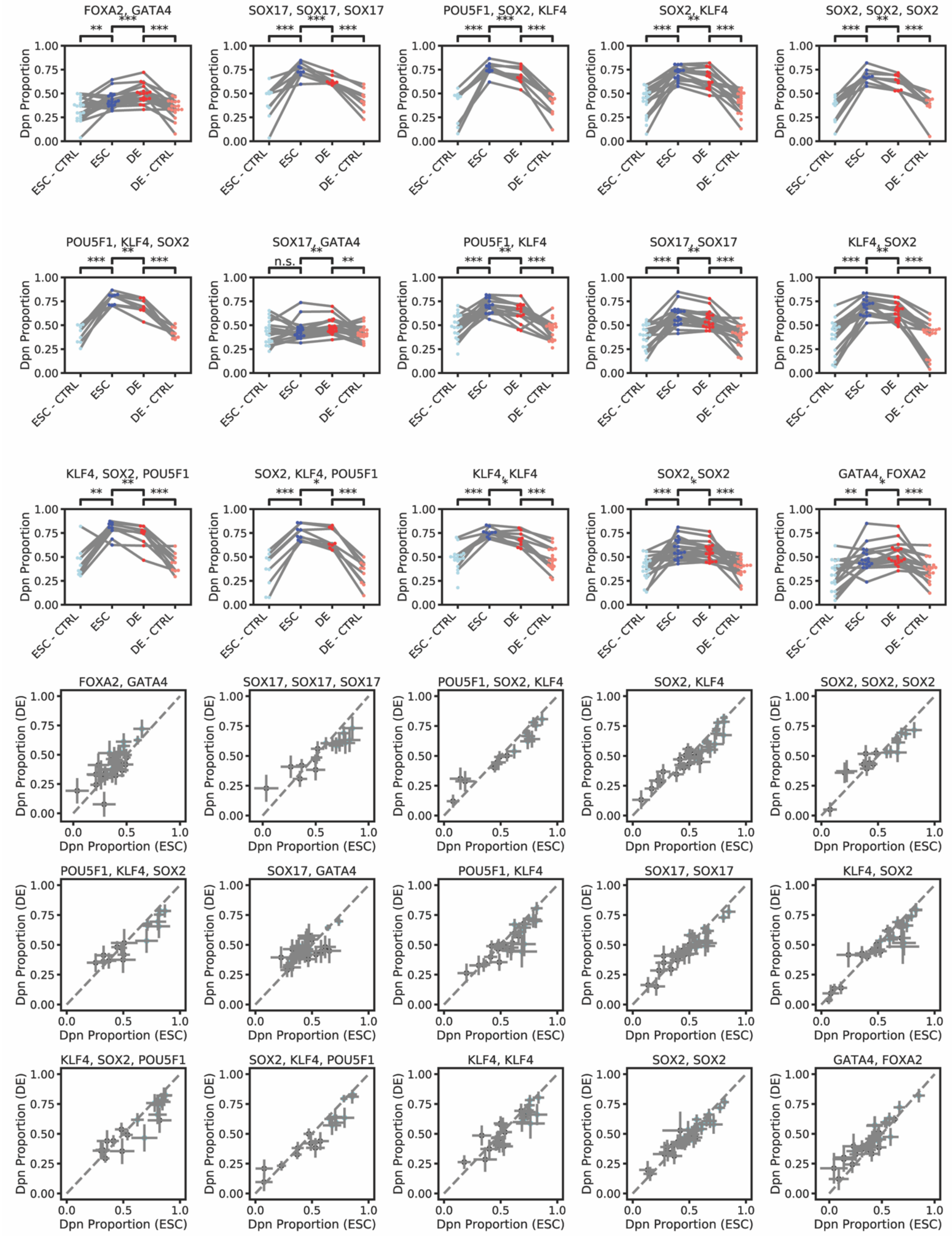

Supplemental Figure S19. All differentially accessible pioneer motifs or combinations of pioneer motifs. 15 out of 42 possible orderings and combinations of 1, 2, or 3 motif sites are

differentially accessible under multiple hypothesis testing. Top shows dot plot of MIAA measured accessibility of each phrase over 9 (1 or 3 motif instances) or 18 (2 motif instances, measuring 2 distances) different sequence backgrounds, and compared to randomly perturbed control sequences. Bottom shows each phrase's MIAA measured accessibility in stem cell (ESC) and definitive endoderm (DE) along with 95% confidence intervals from experimental replicates of MIAA assay.

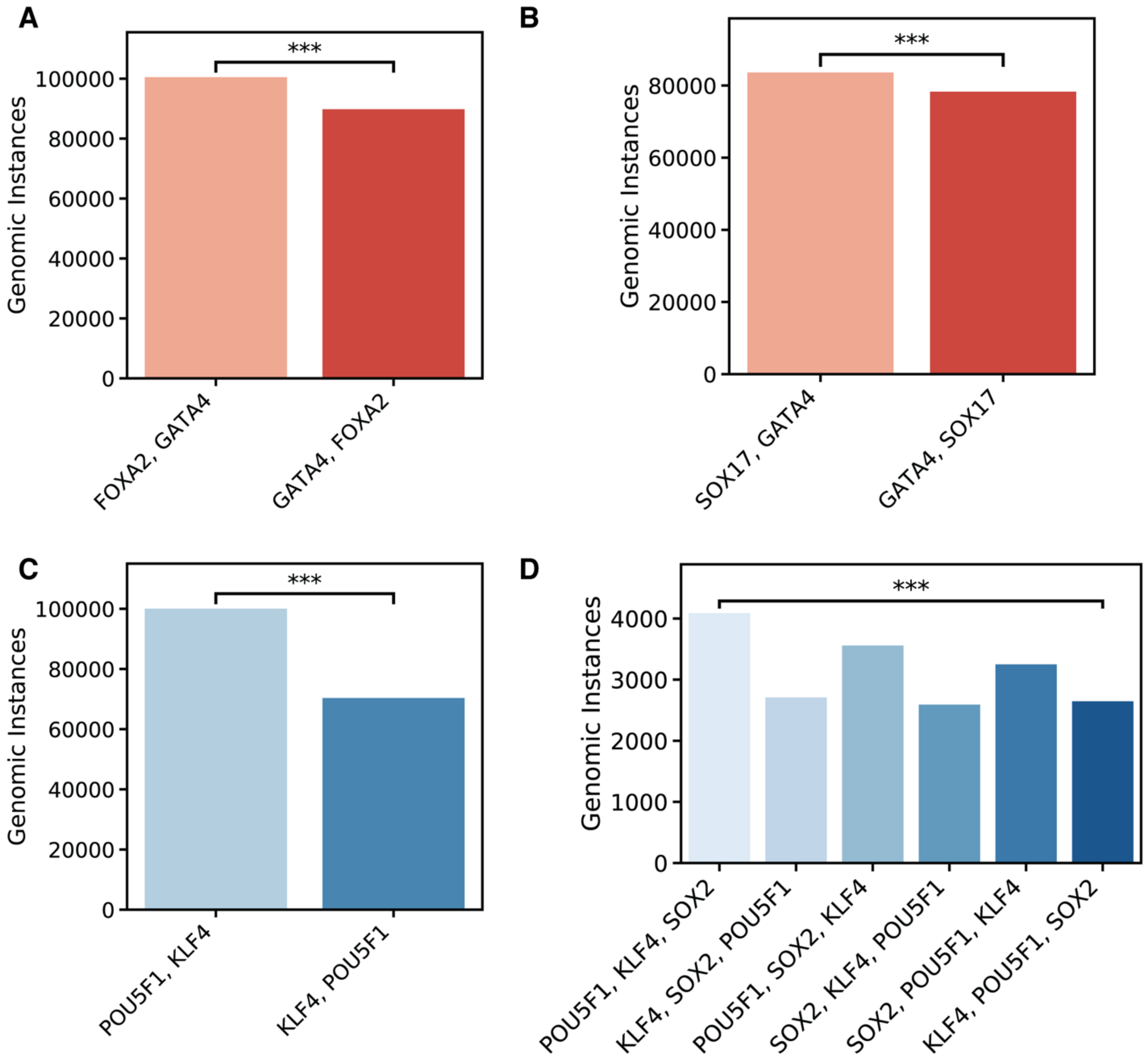

Supplemental Figure S20. Genomic instances of transcription factors reflect ordering for preferential differential accessibility. A) Instances of FOXA2 and GATA4 motifs in 100bp in genome and same orientation as MIAA sequence (+,+). Genome is significantly enriched for instances of GATA4 motif coming second. B) Instances of SOX17 and GATA4 motifs in 100bp in genome and same orientation as MIAA sequence (+,+). Genome is significantly enriched for instances of GATA4 motif coming second. C) Instances of POU5F1 and KLF4 in 100bp in genome and same orientation as MIAA sequence (+,+). Genome is significantly enriched for instances of significantly enriched for KLF4 coming second in the 100bp sequence. D) Instances of POU5F1, SOX2, and KLF4 in 100bp in genome and same orientation as MIAA sequence constructs are significantly enriched for the most differentially accessible order (POU5F1, KLF4, SOX2), but no general trend exists. Significance computed by Chi-Squared test for probability of seeing bias orderings under an expectation of equal probability of each ordering of the transcription factors.

kmer	model	celltype-differential	deepaccess-predicted
GATTGGTTAA-TGTTGTACCG	DeepAccess	TRUE	TRUE
CGCGGCGGCC	DeepAccess	FALSE	N/A
GGTGTGGCC-GCCTTGTTGGAG	KMAC	TRUE	TRUE
GTCACCTGAG	DeepAccess	TRUE	TRUE
ATACCGATAC-GCGCACACCT	DeepAccess	TRUE	TRUE
GGTGTGGCC-CTGCCTG	KMAC	TRUE	TRUE
TGGAGTGACA	DeepAccess	TRUE	TRUE
ATGTTTAC-CGCGGCGGCC	DeepAccess	TRUE	FALSE
TGTTGTACCG	DeepAccess	FALSE	N/A
GATTGGTTAA-GCGCACACCT	DeepAccess	TRUE	TRUE
TGATGTCACT-TGGAGTGACA	DeepAccess	TRUE	TRUE
GGCCATTGT	KMAC	TRUE	TRUE
GATTGGTTAA	DeepAccess	TRUE	FALSE
ACGAATTATC-GTCACCTGAG	DeepAccess	FALSE	N/A
CACCAGGGG	KMAC	FALSE	N/A
TGCAGTGACA-TGGAGTGACA	DeepAccess	TRUE	TRUE
TGTCAACATT-GGAGTGACAC	DeepAccess	TRUE	TRUE
GAGTGACACT-TGTCAACATT	DeepAccess	TRUE	TRUE
ACTCTGT-TGTAACTG	KMAC	FALSE	N/A
TCTGTGTGTGT	KMAC	TRUE	TRUE
TGAGGACCGG	DeepAccess	TRUE	FALSE
ATGTTTAC-GTCACCTGAG	DeepAccess	TRUE	TRUE
CCTCCGCACC-GTAGGTGTTC	DeepAccess	TRUE	TRUE
TGATGTCACT	DeepAccess	TRUE	TRUE
ACGAATTATC-GTGACCACTG	DeepAccess	TRUE	TRUE
TATTGAC	KMAC	FALSE	N/A
CCAGCACCCA-GTAGGTGTTC	DeepAccess	TRUE	FALSE
ATGTTTAC-GTGACCACTG	DeepAccess	TRUE	TRUE
TGCAGTGACA	DeepAccess	TRUE	TRUE
GAGTGACACT	DeepAccess	TRUE	TRUE
TGTAACTG	KMAC	FALSE	N/A
TCCAGTTCCA	KMAC	FALSE	N/A
GGTGTGGCC	KMAC	TRUE	FALSE
CCTCCGCACC	DeepAccess	TRUE	TRUE
GCCTTGTTGGAG	KMAC	FALSE	N/A
ACTGGAACTC-TGCAAATTGT	KMAC	TRUE	FALSE
TATTGAC-TCCAGTTCCA	KMAC	FALSE	N/A
GGAGTGACAC	DeepAccess	TRUE	TRUE
GCGCACACCT	DeepAccess	TRUE	TRUE
TATTGAC-GGCCATTGT	KMAC	FALSE	N/A
CCTCCGCACC-GAACAGGTGC	DeepAccess	TRUE	TRUE
GTGACCACTG	DeepAccess	TRUE	TRUE
GTACCTGTTG	DeepAccess	TRUE	FALSE
ATACCGATAC	DeepAccess	FALSE	N/A
GTCACCTGAG-GTGACCACTG	DeepAccess	TRUE	TRUE
ATGTAAACAT	KMAC	FALSE	N/A
TCTGTGTGTGT-ACTGGAACTC	KMAC	FALSE	N/A
ACGAATTATC-CGCGGCGGCC	DeepAccess	TRUE	FALSE
TGTTTTTGTT	KMAC	FALSE	N/A
GTAGGTGTTC	DeepAccess	TRUE	FALSE
ACGAATTATC	DeepAccess	FALSE	N/A
ATGTTTAC	DeepAccess	FALSE	N/A
ATGTTTAC-ACGAATTATC	DeepAccess	FALSE	N/A
CCAGCACCCA-CGCACACCTG	DeepAccess	FALSE	N/A
GATTGGTTAA-ATACCGATAC	DeepAccess	FALSE	N/A
GTCACCTGAG-CGCGGCGGCC	DeepAccess	FALSE	N/A
CGCGGCGGCC-GTGACCACTG	DeepAccess	FALSE	N/A
CAGCTGTG	KMAC	FALSE	N/A
CTGCCTG	KMAC	FALSE	N/A
TGTTTTTGTT-TGCAAATTGT	KMAC	TRUE	FALSE
GAACAGGTGC	DeepAccess	FALSE	N/A
TGTTTTTGTT-CACCAGGGG	KMAC	FALSE	N/A
CCTCCGCACC-GTACCTGTTG	DeepAccess	FALSE	N/A
CCTCCGCACC-TGAGGACCGG	DeepAccess	TRUE	TRUE
TGTCACCTGA	DeepAccess	TRUE	TRUE
TGCAAATTGT	KMAC	FALSE	N/A
CGCACACCTG	DeepAccess	TRUE	TRUE
TGCAGTGACA-TGATGTCACT	DeepAccess	TRUE	TRUE
ACTCTGT	KMAC	FALSE	N/A
TGTCAACATT	DeepAccess	TRUE	TRUE
ACTGGAACTC	KMAC	FALSE	N/A
ATACCGATAC-TGTTGTACCG	DeepAccess	TRUE	TRUE
TCTGTGTGTGT-CAGCTGTG	KMAC	TRUE	TRUE
GCGCACACCT-TGTTGTACCG	DeepAccess	TRUE	TRUE
TGATGTCACT-TGTCACCTGA	DeepAccess	TRUE	TRUE
CCAGCACCCA	DeepAccess	TRUE	FALSE

Supplemental Table S1. List of 76 motifs and motif pairs tested from DNase-seq motif discovery with KMAC and DeepAccess. Reported motifs that successfully drive differential accessibility

between stem cell and definitive endoderm, and whether those that tested differential were also predicted to be differential from DeepAccess.

Description	Data Type	Accession
Brachyury/Eomes Knock-out mouse ES cells w/ overexpression FoxA2 or Brachyury	RNA-seq	GSE128466
Mouse Stem Cell (d0) and definitive endoderm (d5)	RNA-seq	GSE116259
Brachyury ChIP-seq mesendoderm	ChIP-seq	GSE54978
FoxA2 ChIP-seq definitive endoderm	ChIP-seq	GSE116258
DNase-seq stem cell and definitive endoderm	DNase-seq	GSE53776
Mouse Stem Cell Transcription Factors and H3K27ac	ChIP-seq	GSE92846

Supplemental Table S2. Accession of public data used in analysis.

POU5F1	GGGATGCTAATC
KLF4	GGGGCGGGGCCG
SOX2	GCTCATTGTTTC
SOX17	GCCATTGTTTT
FOXA2	CTTGTTTACATA
GATA4	GCAGATAAGA

Supplemental Table S3. Consensus sequence motifs used for consensus lineage transcription factor MIAA library.
