# Supplementary material for: Identification of determinants of differential chromatin accessibility through a massively parallel genome-integrated reporter assay
Source: Genome Res. 2020 Oct;30(10):1468–80. doi: 10.1101/gr.263228.120 (PMC7605270; doi:10.1101/gr.263228.120)
Supplement: Supplemental Material [file supp_gr.263228.120_Supplemental_Methods.docx]

Table of Contents

[I. Oligonucleotide library integration and NGS library preparation 1](#_Toc47880845)

[II. Rarg-DamN126A sequence 5](#_Toc47880846)

[III. qPCR validation of RAR-DamA126 7](#_Toc47880847)

[IV. Embryonic stem cell line generation, culture, and endoderm differentiation 8](#_Toc47880848)

[V. Previously published genomic data 9](#_Toc47880849)

[VI. DNase-seq processing 9](#_Toc47880850)

[VII. RNA-seq processing 9](#_Toc47880851)

[VIII. ChIP-seq processing 10](#_Toc47880852)

[IX. Brachyury Dimer analysis 10](#_Toc47880853)

[X. KEGG Pathway Analysis of Zeb2 sites 10](#_Toc47880854)

[XI. DeepAccess ensemble architectures 10](#_Toc47880855)

[XII. DeepAccess Stem Cell Reprogramming Factor analysis 11](#_Toc47880856)

[XIII. Statistical test for differential accessibility of k-mers 11](#_Toc47880857)

[XIV. KMAC motif discovery 12](#_Toc47880858)

[XV. Native genomic sequence motif discovery 12](#_Toc47880859)

[XVI. Sequence k-mer motif discovery 12](#_Toc47880860)

[XVII. Linear Regression Models 12](#_Toc47880861)

[XVIII. Genomic motif order enrichment 13](#_Toc47880874)

# Oligonucleotide library integration and NGS library preparation

Oligonucleotide libraries were ordered in the following format from Twist Biosciences:

CACTCAGTACTTTGTCCGTGCTGAC [100-nt sequence] AGATCGGAAGAGCGTCGTGTAGGGA

Note that this sequence includes a reverse complement of the Illumina read 1 sequencing primer to facilitate NGS. All PCR amplification was performed using 2X Q5 UltraII Mastermix (New England Biolabs).

Genomic integration was performed by PCR amplification of oligonucleotide libraries with the following homology arm primers:

RARRXR1 locus (primary locus used in this work)

RARRXR1_library_fw CCTGGTCCAGACACTCATTCTCAAGCTTCCTCATGCTCTTGTGGGAAGCATAGATGCTTTCAGAG CA CTCAGTACTTTGTCCGTGCTGAC

RARRXR1_library_rv CTGTGAGGCTGGTGGAAGACCACAAACAGGGGAGGGTCATGGAGAGGTCAGGGGTTGCCAACAAAGC TCCCT ACACGACGCTCTTCCGAT

uCd8 locus (secondary locus used in this work)

uCd8_library_fw CGAATCACTCCATGTGAGTATCACAGAACGGGTGCAGGAGATCAGTTGCTGTGATGGATAGACACAG TCCCTACACGACGCTCTTCCGAT

uCd8_library_rv ATCCGCCCTGAAGCAGGCAGCAGAGCAGATGCTCTGAGATGCTTGCTTTCTGTAGCCCAGGTGTG CACTCAGTACTTTGTCCGTGCTGAC

for 35 cycles, using 60 second 72 deg extension in each cycle to reduce GC bias. For each 15 cm plate to be electroporated, 1% of the library was amplified in 500 uL PCR volume. MinElute purification with 250 uL PCR product/column was performed, eluting in 10 uL/column and pooling the two columns’ worth of product for each plate to be electroporated.

*Sp*Cas9 gRNAs were cloned into a Tol2-transposon-containing gRNA expression plasmid (Addgene 71485) ^1^.) using BbsI plasmid digest and Gibson Assembly (New England Biolabs). Spacer sequences were as follows:

RARRXR1: GAGCAGGTGACAATTTCAGA

uCd8: GTAGCCCAGGTGTGCAGGCT

For each 15-cm plate to be electroporated, we used 40 ug p2T CBh Cas9 BlastR (Addgene 71489), 40 ug gRNA plasmid, and minElute-purified product from 500 uL PCR.

Electroporations were performed in 2-4 biological replicates into p2L RAR-DamA126 embryonic stem cells by mixing the listed DNA amounts with 5-20*10^6 cells in 300 ul EmbryoMax Electroporation Buffer (Millipore) and electroporating in a 0.4-cm electroporation cuvette using a BioRad electroporator at

230 V, 0.500 mF, and maximum resistance. Electroporated cells were plated in mESC media supplemented with 7.5 mM Y-27632 (Tocris). From 24 to 72 hr after electroporation, media was refreshed daily with mESC media supplemented with 10 ug/ml Blasticidin (Life Technologies) and 66 ug/ml (1:666) Hygromycin (Cellgro). Cells were grown for 5-8 days after electroporation to obtain adequate quantities for Doxycycline treatment and, when indicated, endoderm differentiation followed by Doxycycline treatment.

Genomic DNA library preparation was performed by performing DpnI and DpnII digests using the following conditions:

DpnI digest: 10 ug genomic DNA + 10 uL CutSmart Buffer (New England Biolabs) + 2 uL DpnI (New England Biolabs) + up to 100 uL water.

DpnII digest:20 ug genomic DNA + 20 uL DpnII buffer (New England Biolabs) + 4 uL DpnII 9New England Biolabs) + up to 200 uL water. Reactions were incubated for 16-24 hours at 37 deg then 80 deg for 30 min. Digested genomic DNA product was used directly as input to library prep.

Library prep was performed by 3 successive PCRs. In the first PCR, the entire DpnI-digested genomic DNA was used in a 400 uL PCR rxn, and the entire DpnII-digested genomic DNA was used in a 800 uL PCR rxn. 13 cycles of PCR1 (Q5 UltraII mastermix, Ta=66, 60 second 72 deg extension per cycle) was performed using the following primers:

RARRXR1 locus:

Library_PCR1_fw TCAGTACTTTGTCCGTGCTGAC

RARRXR1_PCR1_rv (for RARRXR1) GTCCACCCTTCCTGTCTGTAC

uCd8 locus:

uCd8_PCR1_fw CCGGTGGGGTCTCAGTGTTAACC

Library_PCR1_rv TCCCTACACGACGCTCTTCCGAT

PCR 1 was purified using PCR purification in a single column, eluting in 50 uL. PCR2 was performed for 7 cycles (Q5 UltraII mastermix, Ta=66, 60 second 72 deg extension per cycle) using 22.5 uL of PCR1 product in a 100 uL reaction with the following primers:

Library_Read1stub ACACTCTTTCCCTACACGACGCTCTTCC

Library_Read2stub GTTCAGACGTGTGCTCTTCCGATCTCAGTACTTTGTCCGTGCTGAC

PCR 2 was purified in a single column, eluting in 50 uL.

qPCR (Q5 UltraII mastermix with EvaGreen (Biotium), Ta=66, 60 second 72 deg extension per cycle) was used to determine PCR3 cycle counts using the following primers:

Read1_noindex AATGATACGGCGACCACCGAGATCTACACTCTTTCCCTACACGACGCTCTTCCGATCT

Read2_noindex CAAGCAGAAGACGGCATACGAGATGTGACTGGAGTTCAGACGTGTGCTCTTCCGATCT

PCR3 was performed by subtracting 2-3 cycles from the Ct and using 2.5 uL of a mix of distinct indexed primers from the NEBNext Dual Index Kit for Illumina (New England Biolabs) using 22.5 uL PCR2 product in a 50 uL reaction volume (Q5 UltraII mastermix with EvaGreen (Biotium), Ta=66, 60 second 72 deg extension per cycle). PCR3 products were PCR-purified and Tapestation (Agilent) was used to quantify and pool samples for NGS.

Illumina Nextseq was used using the following read lengths:

Read 1: 150 nt

Index 1: 8 nt

Index 2: 8 nt

# Rarg-DamN126A sequence

ATG (green); Rarg (purple); DamN126A (blue); V5-His tag (orange); stop codon (red)

ATGGCCACCAATAAGGAGAGACTCTTTGCGCCCGGTGCCCTGGGGCCTGGATCTGGTTACCCAGGAGCAGGCTTCCCATTCGCCTTCCCAGGTGCACTCAGAGGGTCGCCACCATTTGAGATGCTGAGCCCTAGCTTCCGGGGCCTGGGCCAGCCTGACCTCCCCAAGGAGATGGCTTCTCTCTCGGTGGAGACACAGAGCACCAGCTCGGAGGAGATGGTACCCAGCTCTCCCTCACCCCCACCACCTCCTCGGGTCTATAAGCCATGCTTTGTATGCAATGACAAGTCTTCTGGCTACCACTATGGGGTCAGCTCCTGTGAAGGCTGCAAGGGCTTCTTCAGACGCAGCATTCAGAAAAACATGGTGTATACATGTCACCGTGACAAAAACTGTATCATCAACAAGGTCACCAGAAATCGATGCCAGTACTGCAGGCTACAAAAGTGTTTCGAAGTGGGCATGTCCAAGGAAGCTGTAAGGAACGATCGAAACAAGAAGAAAAAGGAGGTAAAAGAGGAGGGCTCGCCCGACAGCTATGAACTGAGTCCACAGTTAGAGGAACTCATCACCAAGGTCAGCAAAGCCCACCAGGAGACTTTTCCCTCACTCTGCCAGCTGGGCAAGTACACCACGAACTCCAGTGCAGATCACCGGGTGCAGCTGGACCTGGGGCTGTGGGACAAGTTCAGCGAGCTGGCCACCAAATGCATCATCAAGATTGTGGAGTTTGCGAAGCGGCTGCCTGGTTTTACAGGGCTCAGCATTGCCGACCAGATCACGCTGCTCAAGGCTGCTTGTCTGGACATCCTAATGCTGCGGATCTGTACAAGGTATACCCCAGAGCAGGACACTATGACATTCTCGGATGGGCTGACCCTGAACCGAACCCAGATGCACAATGCTGGCTTTGGGCCCCTTACAGACCTCGTCTTTGCCTTTGCCGGGCAGCTGCTGCCCCTGGAGATGGATGACACCGAGACTGGGCTACTTAGTGCTATCTGCCTCATCTGTGGAGACCGAATGGACCTGGAAGAGCCCGAGAAGGTGGACAAGCTGCAGGAGCCCCTGCTGGAAGCCCTGAGGCTCTATGCCCGGCGACGGAGACCCAGCCAACCCTACATGTTCCCAAGGATGCTGATGAAAATCACCGACCTCCGGGGCATCAGCACTAAGGGAGCAGAAAGGGCTATAACCCTGAAGATGGAGATTCCAGGCCCGATGCCACCCCTGATCCGAGAGATGCTGGAGAACCCGGAGATGTTTGAGGACGACTCCTCGAAGCCTGGCCCCCACCCCAAGGCTTCCAGTGAGGACGAAGCTCCAGGGGGCCAGGGCAAAAGGGGCCAAAGTCCCCAACCTGACCAGGGGCCCAAGGGTGGGCGCGCCAAGAAAAATCGCGCTTTTTTGAAGTGGGCAGGGGGCAAGTATCCCCTGCTTGATGATATTAAACGGCATTTGCCCAAGGGCGAATGTCTGGTTGAGCCTTTTGTAGGTGCCGGGTCGGTGTTTCTCAACACCGACTTTTCTCGTTACATCCTTGCCGATATCAATAGCGACCTGATCAGTCTCTATAACATTGTGAAGATGCGTACTGATGAGTACGTACAGGCCGCACGCGAGCTGTTTGTTCCCGAAACAAATTGCGCCGAGGTTTACTATCAGTTCCGCGAAGAGTTCAACAAAAGCCAGGATCCGTTCCGTCGGGCGGTACTGTTTTTATATTTGAACCGCTACGGTTACAACGGTCTCTGCCGATACGCTCTGCGCGGTGAGTTTAACGTGCCGTTCGGCCGCTACAAAAAACCCTATTTCCCGGAAGCAGAGTTGTATCACTTCGCTGAAAAAGCGCAGAATGCCTTTTTCTATTGTGAGTCTTACGCCGATAGCATGGCGCGCGCAGATGATGCATCCGTCGTCTATTGCGATCCGCCTTATGCACCGCTGTCTGCGACCGCCAACTTTACGGCGTATCACACAAACAGTTTTACGCTTGAACAACAAGCGCATCTGGCGGAGATCGCCGAAGGTCTGGTTGAGCGCCATATTCCAGTGCTGATCTCCAATCACGATACGATGTTAACGCGTGAGTGGTATCAGCGCGCAAAATTGCATGTCGTCAAAGTTCGACGCAGTATAAGCAGCAACGGCGGCACACGTAAAAAGGTGGACGAACTGCTGGCTTTGTACAAACCAGGAGTCGTTTCACCCGCGAAAAAAGGCGGCGGAGGTAGCGAATTCCCGCGGTTCGAAGGTAAGCCTATCCCTAACCCTCTCCTCGGTCTCGATTCTACGCGTACCGGTCATCATCACCATCACCATTGA

# qPCR validation of RAR-DamA126

Cells were treated with Doxycycline to induce RarDamN126A for 1h and 24h. We performed integration followed by DpnI and DpnII digestion on two “positive control” and two “negative control” regions. The positive controls have strong DNase accessibility and strong RAR binding; the negative controls have neither. qPCR was performed using the following primers:

| Forward Primer | Reverse Primer |
| --- | --- |
| CGCCCTTGCCCTTGGAGAT | AGCTAGCACTCTGGAAATGGC |
| GCAGCCTTTTCAACAACCCTT | TGGGGCAATCAGATTCAAACC |
| CACTGTTCTTTGTGCCATCCCTTTA | TGCTCTGAGATGCTTGCTTTC |
| CTTCTCGCTCCTCCCACTAT | AACAAACACCAGGGCCAGTT |

# Embryonic stem cell line generation, culture, and endoderm differentiation

All experiments were performed in dual LoxP site-containing and rtTA-expressing 129P2/OlaHsd mouse embryonic stem cells (mESC), which were cultured according to previously published protocols^2,3^. Cells were regularly tested for mycoplasma. Cre/LoxP site-specific integration of a Rarg-DamN126A (RAR-DamA126) fusion construct (sequence for the fusion construct is in section XVII) subcloned from a previously published Tcf7l2-DamN126A mutant construct^4^) was performed by electroporation using a previously published protocol^2^ followed by G418 (Life Technologies) selection (350 $\mu$g/mL) for one week to generate the mESC line used in all experiments. To generate sublines with inducible expression of FOXA2 or T, full-length mouse cDNAs for these genes were cloned into a Tol2 expression plasmid containing a 7X TRE promoter and Blasticidin resistance^5^. Co-transfection of Tol2 transposase with TRE-FOXA2/T plasmids was performed followed by selection with 10 ug/mL Blasticidin for one week.

mESCs were maintained on gelatin-coated plates feeder-free in mESC media composed of Knockout DMEM (Life Technologies) supplemented with 15% defined fetal bovine serum (FBS) (HyClone), 0.1mM nonessential amino acids (NEAA) (Life Technologies), Glutamax (GM) (Life Technologies), 0.55mM 2 $\beta$-mercaptoethanol (Sigma), 1X ESGRO LIF (Millipore), 5 nM GSK-3 inhibitor XV and 500 nM UO126. To induce differentiation, cells were grown for 48 hours in mESC media without GSK3 inhibitor XV and UO126 and then seeded at 10^4 cells/cm2 in mESC media without GSK3 inhibitor XV and UO126. After 24 hours, media was replaced with serum-free differentiation media: Advanced DMEM supplemented with N-2, B27 Supplement without vitamin A, and Glutamax (Thermo Fisher). After 24 hours, media was replaced with differentiation media: Advanced DMEM with 2% ES-tested FBS and Glutamax. To induce endoderm differentiation, cells were treated for 24 hours in differentiation media with 50 ng/mL E. coli-derived Activin A (Peprotech) for 24 hours to produce mesendoderm, then cells were fed with Advanced DMEM with 2% FBS, Glutamax, 50 ng/mL Activin A and 1 µM Dorsomorphin (Sigma) for 48 hours. Induction of Rarg-DamN126A expression was performed by treatment of mESCs or mESC-derived endoderm with 2 ug/mL Doxycycline (Sigma) for 24 hours prior to genomic DNA collection using the Purelink Genomic DNA miniprep kit (Life Technologies).

# Previously published genomic data

All previously published data analyzed for this study are listed in Supplementary Table 2.

# DNase-seq processing

DNase-seq reads were aligned to mm10 with bwa aln with the parameters -n 2 -k 2 -t 4. Bams were sorted and paired reads processed with samtools sort and samtools fixmate. Peak accessible regions and signal tracks were generated from aligned reads using the Kundaje lab pipeline (<https://github.com/kundajelab/atac_dnase_pipelines>) using MACS2 for peak calling. For each cell type, we define DNase-seq accessible regions as 100nt regions centered at the MACS2 narrow peak calls from the pooled replicate data. Cell type-specific accessible regions are those which do not physically overlap the other cell type.

# RNA-seq processing

GSE128466 – RNA-seq data was downloaded as fastqs. Adaptors were trimmed with TrimGalore version 0.6.2. Trimmed reads were aligned to mm10 and gene read counts were computed with RSEM^6^ version 1.3.0. GSE116259 – RNA-seq data was downloaded as RSEM quantification files. Differential expression analysis was computed using rsem-run-ebseq and rsem-control-fdr with an fdr threshold of 0.05.

# ChIP-seq processing

ChIP-seq reads were aligned using bowtie2 version 2.2.9 with the parameters –fast. Duplicate reads were removed with samtools rmdup. Binding events were called with GPS version 3.4 with parameters --d $gemdir/Read_Distribution_default.txt --f SAM. Motif analysis was performed using HOMER^7^ findMotifsGenome.pl with parameters -bg brachyury_chip-union.downstream.bed -size 50 -h. Where brachyury_chip-union.downstream.bed is a custom background bedfile of regions immediately downstream of the GPS ChIP binding region. K-mers from sequence library design were tested by first converting k-mers to motifs using HOMER’s^7^ seq2profile.pl, allowing for up to 2 base mismatches.

# Brachyury Dimer analysis

See Supplementary methods ChIP-seq processing for methods for ChIP binding event calling and motif enrichment. After motif enrichment, the Brachyury binding sites were scanned for the top Brachyury motif using HOMER^7^ findMotifsGenome.pl with the parameters -size 50. HOMER^7^ output was processed to identify Brachyury binding regions that consisted of 2 motif instances.

# KEGG Pathway Analysis of Zeb2 sites

ZEB2 motif matches were computed using HOMER^7^ findMotifsGenome.pl on 100nt regions open in stem cells and not definitive endoderm. KEGG enrichment was performed on all Zeb2 motif sites in ESC regions using HOMER^7^ annotatePeaks.pl with the parameters -go $OUTPUT_GO_DIR and otherwise default parameters.

# DeepAccess ensemble architectures

The convolutional filters of the first hidden layer were set to the position weight matrices of the 664 HOMER (Heinz et al. 2010) motifs of known transcription factors. In the ensemble, one convolutional neural network no hidden layers, followed by a global max-pooling layer, representing a logistic regression classifier with motif features. The other 9 convolutional neural networks have a second convolutional layer followed by a global max-pooling layer which allows them to integrate combinatorial and spatial relationships between HOMER motifs.

# DeepAccess Stem Cell Reprogramming Factor analysis

ChIP-seq bed files were downloaded for POU5F1, SOX2, and KLF4, and H3K27ac and uniformly resized to 100nt from the center of the ChIP-seq call. Uniform size peaks were scanned for motif matches, then sequences were generated with motif ablated (randomly shuffle nucleotides within motif sequence) and motif vs motif ablated effects on accessibility in stem cell and definitive endoderm were predicted with DeepAccess.

# Statistical test for differential accessibility of k-mers

For each hypothesis (k-mer or combination of k-mers) a paired t-test and Wilcoxon signed-rank test was computed between the accessibility of sequences in stem cell and accessibility of sequences in definitive endoderm cells. The p-values for each test were adjusted for multiple hypothesis testing by Benjamini-Hochberg procedure with error rate < 0.05. For all sequences which were significant under both t-test and signed rank, a second statistical test was computed within each cell type comparing sequences to paired shuffled controls using paired t-test and Wilcoxon signed-rank test. The p-values for the two cell types were compared, and the lower p-value selected. These tests were adjusted for multiple hypothesis testing by Benjamini-Hochberg procedure with error rate < 0.05, and any hypothesis which passed significance was then considered differentially accessible because it passed both differential between the two cell types as well as differential compared to shuffled controls.

# KMAC motif discovery

To run KMAC, we take the broad pooled peaks as output from MACS2, and select the top 10,000 which are unique (not physically overlapping) in each cell type. We take the top 10,000 peaks that are shared between the two cell types as our background set of sequences. We then run KMAC with the parameters --k_win 100 --k_min 4 --k_max 13 --t 1 --k_seqs 10000 --k_top 10 --gap 4. For the 6,000 sequence library, we selected k-mers from KMAC which showed promise in a pilot MIAA experiment or interesting combinatorial results *in silico* from predictions by our ensemble of convolutional neural networks, DeepAccess.

# Native genomic sequence motif discovery

We ran HOMER^7^ findMotifs.pl with sequences in cluster passed as the positive sequence set, and the shuffled control library sequences for the background sequence set.

# Sequence k-mer motif discovery

To obtain motifs from designed k-mers based on MIAA-measured accessibility in stem cells and definitive endoderm, we first ran agglomerative clustering on the regression weights from the k-mers and k-mer pairs. We then extracted all sequences from a given cluster and ran HOMER^7^ findMotifs.pl with sequences in cluster passed as the positive sequence set, and the same number of randomly selected sequences excluding sequences in the cluster as the background sequence set.

# Linear Regression Models

For visualizing motif accessibility effects, we train linear regression models with the following design matrix, where *r* indicates a replicate-specific offset, *ct* indicates the cell-type indicator, *k* indicates the k-mer or pair of k-mers in the 6k library, and *gc* indicates the GC content of the sequence. Each sequence will have up to 3 non-zero features and no features should be correlated so we apply non-regularized OLS regression.

$$y=\sum_{r} x_{r}\beta_{r}+\sum_{ct} \sum_{k} x_{ct,k}\beta_{ct,k}+{\sum_{ct} \beta_{ct,gc}x}_{ct,gc}$$

# Genomic motif order enrichment

We scanned the whole mouse genome for instances of POU5F1, SOX2, KLF4, SOX17, GATA4, and FOXA2 with the Homer command scanMotifsGenomeWide.pl, using HOCOMOCO motifs due to their uniform length and similarity to the consensus motifs. We then counted instances where two (or three) motifs specific occurred in 100nt on the first strand since all MIAA designed sequences were done with motifs in a (+) orientation. We then performed a Chi-Squared test for the probability of seeing an imbalance for a specific ordering, given the total number of occurrences and assuming the probability of observing a particular ordering is uniform.

**References**

1. Arbab, M., Srinivasan, S., Hashimoto, T., Geijsen, N. & Sherwood, R. I. Cloning-free CRISPR. *Stem cell reports* **5**, 908–917 (2015).

2. Mazzoni, E. O. *et al.* Embryonic stem cell-based mapping of developmental transcriptional programs. *Nat. Methods* **8**, 1056–1058 (2011).

3. Rajagopal, N. *et al.* High-throughput mapping of regulatory DNA. *Nat. Biotechnol.* **34**, 167–174 (2016).

4. Szczesnik, T., Ho, J. W. K. & Sherwood, R. Dam mutants provide improved sensitivity and spatial resolution for profiling transcription factor binding. *Epigenetics Chromatin* **12**, 36 (2019).

5. Sherwood, R. I. *et al.* Discovery of directional and nondirectional pioneer transcription factors by modeling DNase profile magnitude and shape. *Nat. Biotechnol.* (2014) doi:10.1038/nbt.2798.

6. Li, B. & Dewey, C. N. RSEM: accurate transcript quantification from RNA-Seq data with or without a reference genome. *BMC Bioinformatics* **12**, (2011).

7. Heinz, S. *et al.* Simple combinations of lineage-determining transcription factors prime cis-regulatory elements required for macrophage and B cell identities. *Mol. Cell* **38**, 576–589 (2010).
